# High‐Performance Boiling Surfaces Enabled by an Electrode‐Transpose All‐Electrochemical Strategy

**DOI:** 10.1002/advs.202413142

**Published:** 2024-12-25

**Authors:** Yu‐Ming Chen, Nan Hu, Jia‐Yi Zhang, Yi‐Fei Sun, Yue‐Fei Wu, Zi‐Rui Li, Li‐Wu Fan

**Affiliations:** ^1^ Institute of Thermal Science and Power Systems School of Energy Engineering Zhejiang University Hangzhou 310027 China; ^2^ Department of Mechanical and Aerospace Engineering Princeton University Princeton NJ 08544 USA; ^3^ Department of Mechanical Science and Engineering University of Illinois at Urbana‐Champaign Urbana IL 61801 USA; ^4^ State Key Laboratory of Clean Energy Utilization Zhejiang University Hangzhou 310027 China

**Keywords:** critical heat flux, dendritic structures, electrochemical treatment, heat transfer coefficient, pool boiling heat transfer

## Abstract

High‐performance boiling surfaces are in great demand for efficient cooling of high‐heat‐flux devices. Although various micro‐/nano‐structured surfaces have been engineered toward higher surface wettability and wickability for enhanced boiling, the design and fabrication of surface structures for realizing both high critical heat flux (CHF) and high heat transfer coefficient (HTC) remain a key challenge. Here, a novel “electrode‐transpose” all‐electrochemical strategy is proposed to create superhydrophilic microporous surfaces with higher dendrites and larger pores by simply adding an electrochemical etching step prior to the multiple electrochemical deposition steps. Enabled by the high nucleation density and high wicking capability, a high boiling performance is shown on such “etching‐then‐deposition” surfaces with simultaneously high CHF of 2,641 ± 10 kW m^−2^ and high HTC of 214 ± 6 kW (m^2^ K)^−1^, which are more than 2.5 and 4.3‐fold enhanced from those on smooth surfaces, respectively. A very stable morphology and boiling performance of such surfaces subject to consecutive tests are also shown. Using this strategy, such superhydrophilic microporous layers are fabricated on curved surfaces with larger areas, both on spheres and slender cylinders, and demonstrate excellent boiling performance in quenching tests. This facile, geometry‐adaptive, durable, and scalable strategy is very promising for making high‐performance boiling surfaces for large‐scale industrial applications.

## Graphical abstract

An all‐electrochemical strategy with an electrode‐transpose step is proposed to fabricate superhydrophilic microporous surfaces with longer dendrites and larger pores, thus enabling excellent boiling performance with both high critical heat flux and high heat transfer coefficient. This facile, geometry‐adaptive, durable, and scalable strategy has great potential for making high‐performance boiling surfaces for large‐scale industrial applications.

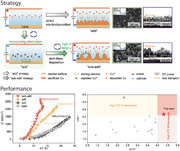

## 1 Introduction

As the exponentially growing need for high‐performance computing, efficient energy storage and utilization, high‐power‐density electronic devices, electrochemical batteries, and chemical/nuclear reactors are being developed to achieve greater compactness, integration, and power density. This progress offers new opportunities for scientific research and engineering applications while imposing higher demands on heat dissipation capabilities.^[^ ^1^, ^2^, ^3^, ^4^, ^5^ ^]^ By virtue of its significant latent heat absorption through vaporization, boiling heat transfer has been extensively studied and utilized as an effective solution for thermal management in high‐heat‐flux devices, offering superior heat flux and dissipation rates compared to single‐phase heat transfer.^[^ ^6^ ^]^ The critical heat flux (CHF) and heat transfer coefficient (HTC) are key parameters used to characterize boiling heat transfer performance.^[^ ^7^, ^8^ ^]^

Consequently, enhancing both CHF and HTC of heating surfaces has emerged as a pivotal topic in the heat transfer community in recent years.^[^ ^9^, ^10^, ^11^, ^12^, ^13^ ^]^ Studies have demonstrated that surfaces with more nucleation sites activated early during nucleate boiling can achieve higher heat flux with lower wall superheat, thus enhancing the HTC. Surfaces having excellent wickability improve liquid replenishment, suppressing dry spot/patch propagation and delaying CHF occurrence.^[^ ^14^, ^15^, ^16^ ^]^ In the meantime, the optimal interplay of liquid supply and bubble departure is also a main interest for surface design.^[^ ^17^, ^18^, ^19^ ^]^ These insights have provided more theoretical support for drastically enhancing the boiling heat transfer using various micro/nano‐fabrication techniques to create multi‐tier structure features, such as nanopores,^[^ ^20^, ^21^, ^22^, ^23^, ^24^ ^]^ nanowires,^[^ ^25^, ^26^, ^27^ ^]^ and microchannels,^[^ ^28^, ^29^, ^30^, ^31^, ^32^ ^]^ on boiling surfaces.

There are numerous approaches to fabricating hierarchical surfaces, which can generally be categorized into three main types: top‐down subtractive manufacturing, bottom‐up additive manufacturing, and hybrid methods that combine both. Top‐down techniques, such as photolithography, computerized numerical control (CNC) machining, and ion etching, offer precise control over the creation of molded structures.^[^ ^33^, ^34^ ^]^ However, these methods are often complex, expensive, and typically suited to flat surfaces, which limits their scalability for large‐scale applications. In contrast, bottom‐up methods, such as physical deposition (including vapor‐phase deposition and self‐assembly) and electrochemical deposition, can form effective hierarchical surfaces despite relatively random structures at low cost and low complexity.

**Figure 1:**
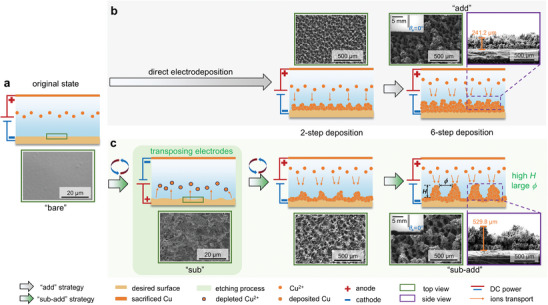
The schematic illustrates the procedures for different ECD strategies. a) original state. b) the conventional direct electrodeposition strategy to fabricate “add” surface, which has been widely adopted by the heat transfer community for preparing superhydrophilic microporous boiling surfaces. In contrast, c) the novel “electrode‐transpose” all‐electrochemical strategy proposed in this work, involves simply transposing the electrodes to introduce a pre‐etching step before proceeding with the deposition steps. This strategy allows “sub‐add” surfaces with a larger dendrite height (*H*) and bigger hole diameter (*ϕ*) than “add” surface, resulting in higher surface roughness, stronger liquid replenishment capability, and less thermal resistance for enhancing boiling heat transfer performance.

Among the various surface fabrication techniques, electrochemical deposition (ECD) stands out due to its advantages, such as low cost, ease of operation, and scalability. To date, ECD has been well‐developed to form porous coatings with regularly patterned microscale pores and irregular dendritic structures. These features provide excellent wickability and numerous nucleation sites, simultaneously enhancing CHF and HTC.^[^ ^35^, ^36^ ^]^ Various studies have investigated the effects of different parameters during the deposition procedure on the morphology of porous coatings and their pool boiling heat transfer performance (see Section S1, Supporting Information). However, as compared to other surface modification methods (e.g., lithography, see Section S1, Supporting Information), the boiling heat transfer performance attained is relatively low with the ECD‐fabricated surfaces because the traditional ECD method cannot precisely control the surface morphology. As shown in **Figure** **1**a,b, due to the smoothness of the bare copper surface, Cu^2+^ particles are prone to uniformly deposit onto the cathode, resulting in an undesirable Cu deposition layer on the substrate. This layer of Cu particles can introduce extra thermal resistance on the surfaces, which is detrimental to the enhancement of HTC.

Manipulating the morphology of electrodeposition has been extensively investigated in the field of electrochemical energy storage, especially the dendrite growth control on the electrode of Li‐ion batteries.^[^ ^37^, ^38^, ^39^, ^40^ ^]^ Previous research has shown that by intentionally controlling the electro‐etching process before the growth of electrodeposition, the growth behavior of the crystals during the deposition process can be effectively regulated.^[^ ^41^, ^42^ ^]^ Hence, to alleviate the drawbacks of the “add” surfaces in Figure 1b, we proposed a multi‐step, “electrode‐transpose” all‐electrochemical strategy. It simply integrated both electrochemical etching and deposition techniques to form controllable dendritic structures on pre‐etched surfaces. Initially, an electrochemical etching procedure was added to roughen the originally smooth bare surface before applying regular electrochemical deposition. As shown in Figure 1c, by first transposing the anode and cathode of the power supply to create an electro‐etched rough substrate, Cu^2^⁺ particles can accumulate at the tips of etched morphology, leaving the basin parts with a thinner layer of deposited Cu. This “electrode‐transpose” manufacturing process resulted in surfaces with higher dendrites, larger holes, and thinner bottom layers. Such features facilitated surface liquid replenishment and bubble departure without increasing thermal resistance, which is beneficial for boosting boiling heat transfer performance in terms of both CHF and HTC.

## 2 Results and Discussion

The microscale morphology of the fabricated “sub‐add” and “add” surfaces (detailed procedure seen in Experimental Section) is shown in Figure 1c,b, respectively. By taking top‐view and side‐view  images by scanning eletron microscope (SEM), we analyzed the representative pore size *ϕ* and dendrite height *H* of both surfaces. We found that a pore size *ϕ* ranges from 35 to 55 µm for the “add” surface and 65 to 85 µm for the “sub‐add” surface, and a dendrite height *H* ranges from 240 to 310 µm for the “add” surface and 400 to 530 µm for the “sub‐add” surface. The pore size for each surface was estimated based on ten holes in two top‐view SEM images, while the average dendrite height was based on ten dendrites in two side‐view SEM images. As expected, we achieved a larger average pore size and a higher dendrite height on the “sub‐add” surface.

**Figure 2:**
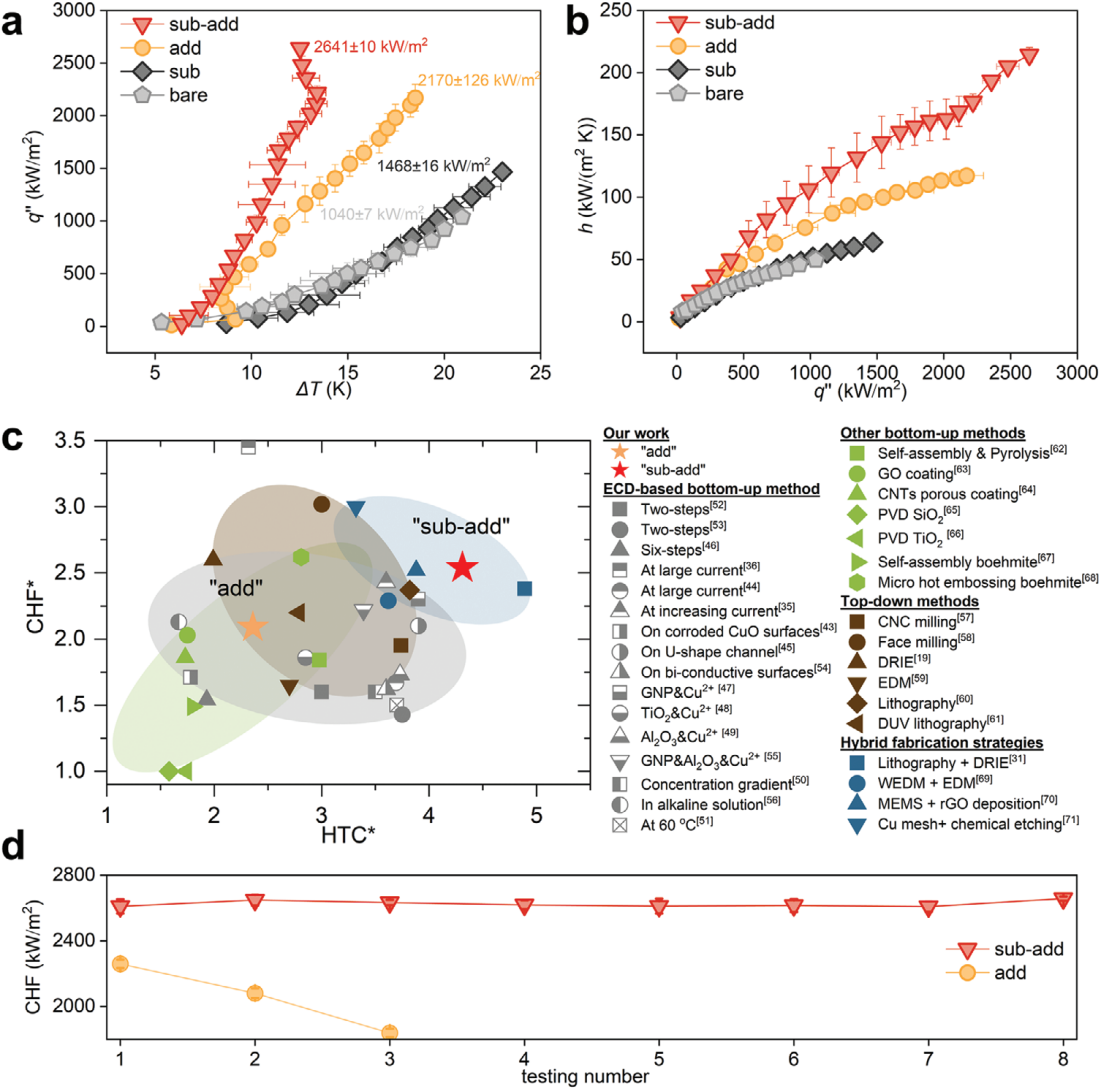
Pool boiling heat transfer performance and durability tests. a) Pool boiling curves and b) HTC as a function of heat flux for four types of surfaces. The “sub‐add” surface shows the best performance at all stages of the boiling process, achieving the highest CHF ≈2641 kW m^−2^ and maximum HTC of ≈214 kW (m^2^ K)^−1^ among the four types of surfaces, which are enhanced up to 254% and 431% compared to bare surfaces, respectively. c) Comparison of the maximum HTC and CHF with related works, where HTC^*^ represents the ratio of the maximum HTC achieved divided by the maximum HTC of the bare surface in the corresponding work, and CHF^*^ represents the ratio of the maximum CHF of the modified surfaces divided by the CHF of the bare surface in the corresponding work. Only the “sub‐add” surface in this work showed extremely high HTC and CHF enhancement ratios simultaneously. d) Durability test results of the CHF value on the “add” and “sub‐add” surfaces. The consecutive tests were conducted on the same surface with a time interval of one week. Abbreviations: GNP, graphene nanoparticles; GO, graphene oxide; CNT, carbon nanotubes; PVD, physical vapor deposition; CNC, computerized numerical control; DRIE, deep reactive ion etching; DUV, deep ultraviolet; WEDM, wire electrical discharge machining; EDM, electrical discharge machining; MEMS, micro‐electro‐mechanical system; rGO, reduced graphene oxide.

The steady‐state pool boiling heat transfer experiments were conducted on a well‐controlled test platform (illustrated in Figure S4). The experimental data for the bare copper surface is repeatable and consistent with the classic models (Section S3, Supporting Information). Heat flux and heat transfer coefficient for the four types of surfaces (see Experimental Section) are presented in **Figure** **2**a,b. As shown in Figure 2a, the boiling curve for the “sub” surface does not present a significant difference from the bare copper surface, whereas the heat flux of the “add” and “sub‐add” surfaces far exceeds that of the bare surface at the same wall superheat. Specifically, when the wall superheat is at 12 K, the heat flux on the “add” surface is close to 1200 kW m^−2^ and reaches ≈2000 kW m^−2^ on the “sub‐add” surface, while the bare surface is only ≈400 kW m^−2^. Compared to the bare surface, CHF increases by ≈41% on the “sub” surface and 109% on the “add” surface, reaching 1468 and 2170 kW m^−2^, respectively. Benefiting from the combination of etching and deposition processes, the CHF on the “sub‐add” surface is further improved to 2641 kW m^−^^2^, which is 154% higher than that of the bare surface. In terms of HTC curves (Figure 2b), the “add” surface shows much higher HTC compared to both the bare and “sub” surfaces, while the HTC curve of the “sub‐add” surface is even higher than that of the “add” surface. The maximum HTC of the “sub‐add” surface is able to reach up to ≈214 kW (m^2^ K)^−1^, 331% higher than that of the bare surface.

To further illustrate the superior performance of the “sub‐add” surface mentioned above, results of related works (saturated pool boiling with water as working fluid, 1 atm) were compared with this work and depicted in Figure 2c.^[^ ^18^, ^31^, ^35^, ^43^, ^44^, ^45^, ^46^, ^47^, ^48^, ^49^, ^50^, ^51^, ^52^, ^53^, ^54^, ^55^, ^56^, ^57^, ^58^, ^59^, ^60^, ^61^, ^62^, ^63^, ^64^, ^65^, ^66^, ^67^, ^68^, ^69^, ^70^, ^71^ ^]^ For convenience, we used HTC^*^ to represent the ratio of the maximum HTC achieved to the maximum HTC of the bare surface in the corresponding work and CHF^*^ in a similar manner for describing the relative improvement in CHF. Compared to other works employing various surface modification strategies, including “bottom‐up,” “top‐down” and hybrid fabrication strategies, our “sub‐add” surface with HTC^*^ of 4.31 exhibits simultaneously high HTC and CHF enhancement, occupying a favorable position among electrochemical‐only methods (see Section S1 for detailed information, Supporting Information). Additionally, as shown in Figure 2d, the “sub‐add” surface exhibits good durability, maintaining a stable CHF value across eight continuous pool boiling tests, with the time interval between two consecutive tests being 1 week. In contrast, the durability of the “add” surface is poor, showing a significant drop in its CHF value during the course of only three tests. The SEM images confirm that there are no obvious changes in its microscale morphology for the “sub‐add” surface before and after the durability tests (See Section S6, Supporting Information).

**Figure 3:**
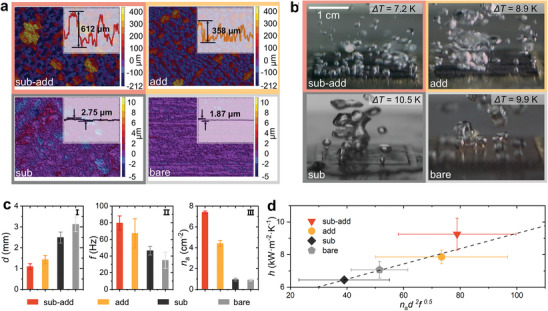
HTC enhancement mechanism at a low heat flux (taking 70 kW m^−^^2^ as an example). a) Distinct morphology characteristics for four surfaces (captured by a three‐dimensional interference microscope). b) Snapshot of arising bubble captured by a high‐speed camera at 500 fps for four surfaces. c) Statistical analyses of nucleation site density *n*_a_, average bubble detachment frequency *f*, and average bubble departure diameter *d* on the four types of surfaces. d) A scatter plot of HTC as a function of *n*_a_ *d* ^2^ *f* ^0.5^ at this stage obtained for the four surfaces based on Rohsenow's HTC model. The “sub‐add” surface demonstrated characteristics of having the largest average pore size and the highest vertical structural differences that rendered the bubble behavior of this surface characterized with smallest average bubble detachment diameter, highest average bubble detachment frequency, and largest nucleation site density, which led to the highest HTC at this stage.

Morphology characterization (see **Figure** **3**a) on the four types of surfaces and the high‐speed imaging for capturing bubble behaviors at a heat flux of ≈70 kW m^−2^ (see Figure 3b) clarify the mechanisms governing the extreme HTC enhancement at low heat fluxes on the “sub‐add” surface. The classical correlation of HTC in the initial stage of nucleate boiling, HTC ~ *n*_a_ *d* ^2^ *f* ^0.5^, suggests that a boiling surface having a higher nucleation site density (*n*_a_), higher average bubble departure frequency (*f*), and larger bubble departure diameter (*d*) is able to reach a higher HTC.^[^ ^72^ ^]^ As depicted in Figure 3a, the interconnected dendritic structures on both the “add” and “sub‐add” surfaces form a honeycomb‐like porous architecture, which increases surface roughness and specific surface area, leading to an increasing number of activated nucleate sites (see Figure 3b). These structures also facilitate the bubble departure, reducing the bubble departure diameter and increasing the bubble departure frequency (see Figure 3b and bubble departure videos in Section S11, Supporting Information). Section S7 (Supporting Information) includes a more detailed explanation of the mechanism based on the bubble growth force model. We statistically analyzed the nucleation site density (*n*_a_), average bubble departure frequency (*f*), and average bubble departure diameter (*d*) for the four types of surfaces (see Figure 3c) and examined the relationship between the experimentally obtained HTC values under low heat fluxes and the *n*_a_ *d* ^2^ *f* ^0.5^ term. As shown in Figure 3d, the HTC values of the four types of surfaces are approximately proportional to the *n*_a_ *d* ^2^ *f* ^0.5^ term, indicating that despite the “sub‐add” surface having the smallest average bubble departure diameter, its higher nucleation site density and higher average bubble departure frequency enable its best boiling heat transfer performance among the four surfaces at the low heat flux.

**Figure 4:**
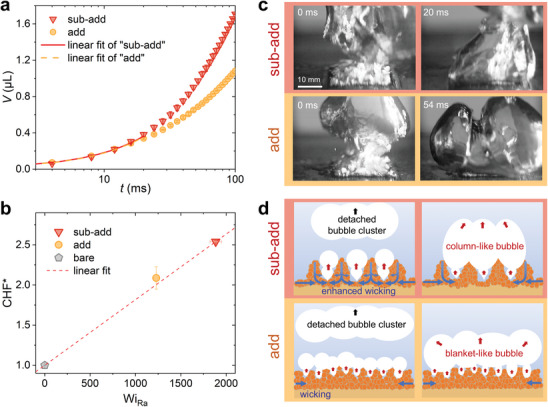
HTC and CHF enhancement mechanism at a high heat flux (taking 1900 kW m^−^^2^ as an example). a) The curve of absorbed liquid volume over time from 0 to 100 ms for the “add” and “sub‐add” surfaces. b) Plots of CHF^*^ over Wi_Ra_ for the bare, “add,” and “sub‐add” surfaces, Wi_Ra_ is the wicking number Wi modified by surface roughness.^[^ ^76^ ^]^ c) Snapshots and (d) schematics of bubble behaviors on the “sub‐add” and “add” surfaces, showing enhanced bubble departure dynamics and liquid replenishment capability due to the manipulated dendritic structures.

As mentioned above, apart from the high HTC at low heat fluxes, the CHF and HTC at high heat fluxes on the “sub‐add” surface are also remarkably high. Slightly different from the mechanisms governing the nucleate boiling heat transfer at low heat fluxes, the boiling heat transfer is dominated by the interactions between the vapor/liquid and the boiling surface at high heat fluxes. Although several different models (bubble interference model, hydrodynamic instability model, macrolayer dryout model, hot/dry spot model, and interfacial lift‐off model) have been developed over the past few decades to describe these interactions and consensus has yet to be reached, their basic understandings are almost identical.^[^ ^73^ ^]^ The heat transfer performance at this stage is determined by the extent of the surface to be rewetted by the bulk liquid for continued nucleation. For instance, in the hydrodynamic instability model, CHF is triggered by the coalescence of adjacent vapor jets driven by growing Helmholtz instability, forming a vapor mushroom on the boiling surface that impedes further liquid rewetting.^[^ ^73^ ^]^ In the hot/dry spot model, CHF occurs due to the irreversible expansion of dry spots on the boiling surface, which prevents further wetting.^[^ ^73^ ^]^ Therefore, previous research typically attributed the higher HTC and CHF on a surface with micro/nanostructures to enhance wickability.^[^ ^31^, ^74^ ^]^ On this basis, we first performed capillary wicking tests on the “add” and “sub‐add” surfaces by liquid‐level‐drop method^[^ ^17^ ^]^ (see Section S8, Supporting Information), and the results are given in **Figure** **4**a. Since the liquid replenishment process is instantaneous at a high heat flux, the absorption volume obtained at the beginning of wicking tests is usually considered to affect HTC and CHF, whereas the following absorption volume change, influenced by the saturated absorption capability of the surface, cannot provide clear evidence for the enhancement of HTC and CHF.^[^ ^74^ ^]^ However, the absorption volume and the initial wicking rate *U*′ within the first 20 ms for the “sub‐add” surface don't have a significant increase compared to the “add” surface, which can be attributed to the heterogeneity of coating structures (see Section S8, Supporting Information).

In view of the higher dendritic structures on the “sub‐add” surface compared to that on the “add” surface, liquid and vapor pathways can be more effectively manipulated and segregated.^[^ ^75^ ^]^ This configuration reduces the liquid supplement resistance feeding to the bubble column base, mitigates bubble coalescence near the surface, and delays the growth of dry areas. Hence, a modified wicking model was adopted,^[^ ^76^ ^]^ and the surface roughness Ra was selected to quantify the above effects of higher dendrites^[^ ^76^, ^77^ ^]^ to obtain the roughness‐modified wicking number (Wi_Ra_) that is defined by the product of the wicking number and the surface roughness ratio (see Section S8, Supporting Information). The relationship between Wi_Ra_ and CHF* is plotted in Figure 4b, where the good positive linear correlation suggests that the extremely high boiling heat transfer performance of the “sub‐add” surface under high heat flux is attributed to the higher dendritic structures.

Meanwhile, as shown in Figure 4c, the high‐speed images of the bubble evolution on both the “add” and “sub‐add” surfaces at a high heat flux of ≈1900 kW m^−2^, which approaches the CHF of the “add” surface but is lower than that of the “sub‐add” surface, also support the above observations. On the “add” surface, bubble columns coalesce horizontally and are stuck on the top of the surface, forming a blanket‐like bubble cluster onto the surface (see Figure 4c,d). In contrast, the “sub‐add” surface exhibits a totally different bubble behavior. Although horizontal coalescence still occurs, the coalesced bubble cluster has a higher upward momentum, resulting in a column‐like bubble cluster (see Figure 4c,d). Similar differences can also be observed at other high heat fluxes (see Section S9 and Videos in Section S11, Supporting Information). This corroborates that the liquid replenishment capability and the bubble departure frequency of the “sub‐add” surface are indeed enhanced by its larger dendritic structure compared to the “add” surface, as illustrated in Figure 4d.

**Figure 5:**
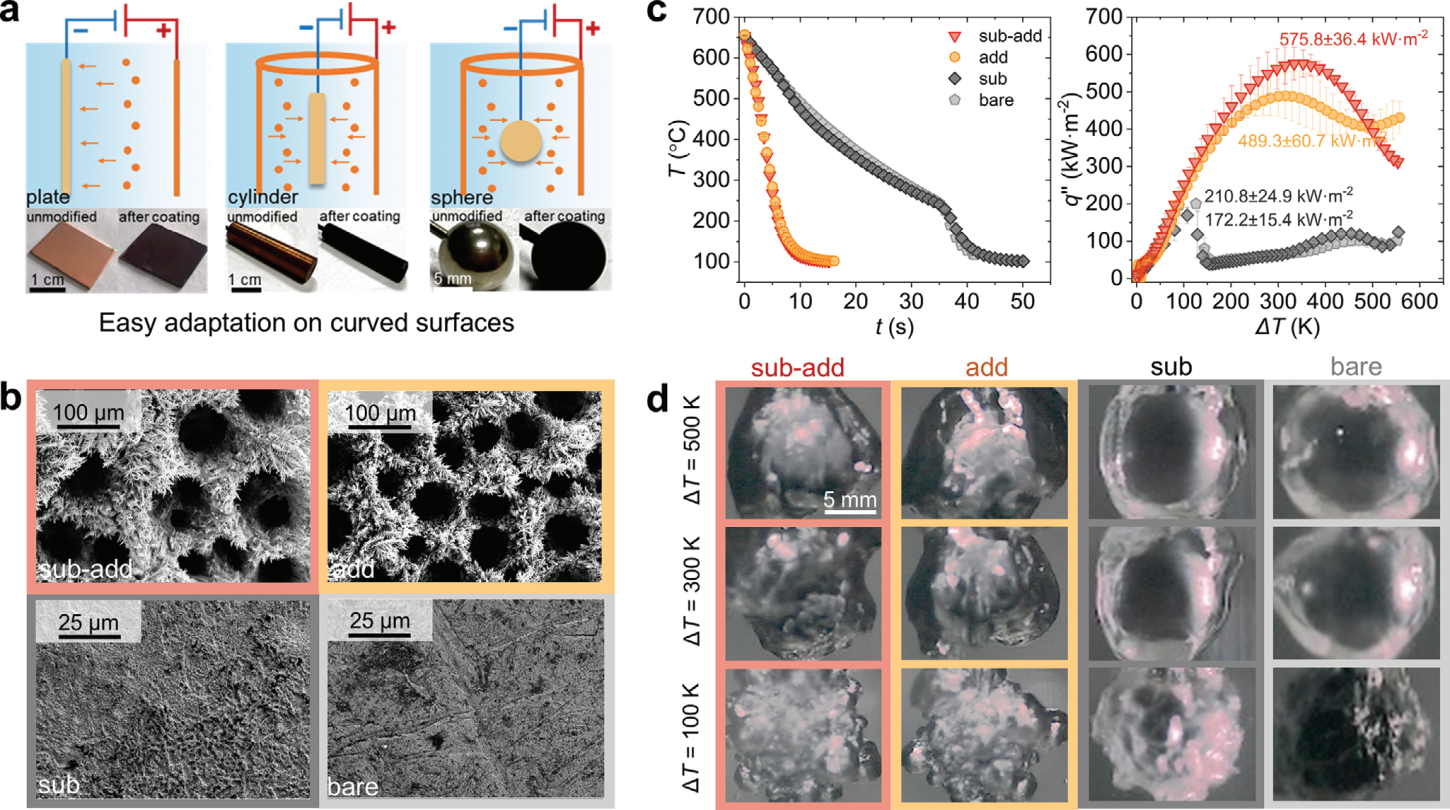
Additional quenching tests of the “electrode‐transpose” all‐electrochemical strategy on curved surfaces. a) Surface fabrication on plates, cylinders, and spheres, showing a wide curvature‐adaptability. b) Four surface morphologies on stainless steel spheres by different modification strategies (captured by SEM). c) The evolution of center temperatures over time and boiling curves, and d) corresponding bubble behaviors during quenching for four surfaces. Compared with other surface modification methods, electrochemical deposition can be easily adapted onto curved surfaces. Quenching experiment results demonstrated that compared with the “add” surface, the “sub‐add” surface also showed better transient boiling heat transfer performance, further demonstrating the reliability and applicability of this surface modification strategy.

In order to further validate the feasibility and applicability of this “sub‐add” strategy onto curved surfaces, we applied the same electrochemical deposition method to construct porous structures on 304 stainless steel spheres. As shown in **Figure** **5**a, compared with well‐structured surfaces from works using other techniques, surfaces modified by the ECD method can be easily adapted to curved surfaces by simply changing the shape of the anode. Figure 5b depicts the (SEM) images of four types of ECD spheres. The details of surface fabrication can be seen in Section S10 (Supporting Information). From Figure 5b, it can be seen that the bare surface is relatively smooth, with some minor scratches. In contrast, the “sub” surface has more pits, and its overall appearance is rougher compared to the bare surface. Both the “add” and “sub‐add” surfaces show a honeycomb‐like porous structure, with finer dendritic crystals growing on the porous scaffolds. Compared to the “add” surface, the average diameter of the pores on the “sub‐add” surface is slightly larger, and the depth of the pores increases. Therefore, it is apparent that when performing electrodeposition on a spherical surface, by adding an electrochemical etching step before electrodeposition, surface morphology can also be controlled to a certain extent.

Subsequently, quenching experiments were conducted on the four types of surfaces by using the ball geometry. The details of the experimental setup and testing principles are included in Section S10 (Supporting Information). The results are shown in Figure 5c. Although the heat flux and cooling rate of the “sub” surface is smaller than the bare surface, the BHT performance of the “sub‐add” surface is still better than the “add” surface. In terms of the overall quench cooling rate, the cooling rate of the “sub‐add” surface is slightly faster than that of the “add” surface. Except in the range where the wall superheat is higher than 500 K, where the boiling curve of the “add” surface is above that of the “sub‐add” surface, and at stages where the wall superheat is less than 500 K, the heat flux of the “sub‐add” surface is consistently higher than that of the “add” surface. The CHF value of the surface also increases by ≈90 kW m^−2^ compared to the “add” surface. Therefore, it can be deduced that, under the same deposition procedure, by adding an electro‐etching step prior to deposition, the quench rate of the stainless‐steel ball surface increases, and the transient boiling heat transfer performance is better. It is worth noting that the superior performance of the “sub‐add” surface could be further enhanced by optimizing the operating conditions of fabrication because here an almost identical procedure was adopted, though the material and geometry of the substrate were different.

Figure 5d presents some vapor film evolution images captured by a high‐speed camera during the transient boiling experiments. A stable vapor film still covers “sub” and bare surfaces at the wall superheat of 300 K. The vapor film rupture on both surfaces is not apparent until the wall superheat is ≈100 K, and the boiling then transfers to nucleate boiling. In contrast, the intense perturbations of the vapor film on two deposited surfaces start even at the wall superheat of 500 K. This suggests that the transition from stable film boiling to nucleate boiling has already been initiated at this wall superheat. As the wall superheat keeps decreasing more intense perturbation eventually triggers the collapse of the vapor film. The vapor film evolution diagram during the boiling process further indicates that the existence of the porous core absorption structure on the two deposited surfaces significantly enhances the transient boiling heat transfer performance of the surfaces.

## 3 Conclusion

In this work, we proposed an “electrode‐transpose” all‐electrochemical strategy to alter the morphology of electrodeposited surfaces for superior boiling heat transfer performance. By simply adding one step of electrochemical etching before deposition, the “sub‐add” surface exhibited a different morphology with higher dendrites and larger pores. The CHF value of the “sub‐add” surface could reach 2641 kW m^−^^2^ (154% higher than the bare surface), which was significantly higher than the “add” surface (2170 kW m^−^^2^). Compared to other related works based on electrochemical deposition, the “sub‐add” surface in this study achieved both excellent HTC and CHF enhancement. In the low heat flux region of the nucleate boiling, an increased number of activated nucleation sites and a higher bubble detachment frequency significantly enhanced the boiling heat transfer performance of the “sub‐add” surface. At high heat flux stages, the larger dendritic structure on the “sub‐add” surface improved the liquid replenishment capability, mitigated the large bubble coalescence near the surface, and accelerated vapor detachment from the surface. This allowed the surface to withstand a higher heat flux, resulting in an increased CHF value. Additionally, quenching experiment results on curved surfaces showed that the “sub‐add” surface also had the best transient boiling heat transfer performance, further demonstrating the reliability and applicability of this surface modification strategy. This strategy, which simply adds one etching step by swapping the electrodes before deposition, offers the advantages of low cost, ease of scalability, and the potential for further enhancement by altering the parameters of both the “sub” and “add” procedures, thus holding great potential for making high‐performance boiling surface toward large‐scale industrial applications.

## 4 Experimental Section

### Surface Fabrication

In this work, 25 mm × 25 mm × 1 mm copper sheets was used as raw materials. Each surface first went through a cleaning procedure: immersed into 1 mol L^−1^ HCl solution for 5 min to remove the oxide layer; carefully polished with #400, #1000, and #2000 sandpaper in turn; placed into ethanol solution and washed in an ultrasonic bath for 15 min to remove organic impurities attached to the surfaces. Electrochemical treatment was performed after the cleaning procedure above. A two‐electrodes electrochemical system was used for surface modification. A 30 mm × 30 mm × 1 mm copper surface was used as an anode and a 25 mm × 25 mm × 1 mm one as the cathode. The anode was slightly bigger than the cathode for the sake of uniform deposition on the cathode. The electrode distance was 30 mm and the system was placed at room‐temperature (≈25 °C). To construct hierarchical porous structures for “add” surfaces, a six‐step deposition procedure was utilized, and the current was set constant during each step. A high current density (*I*_1_) was first applied for a short time to form dendrite structures followed by a very low current density (*I*_0_) for a long time. Such a process was repeated three times in total. The optimized parameters of deposition were set as: *I*_1_ = 1 A cm^−2^, Δ*t*_1_ = 30 s, *I*_0_ = 0.02 A cm^−2^, Δ*t*_0_ = 2500 s, and the electrolyte was the solution of 0.2 mol L^−1^ CuSO_4_ with 1.0 mol L^−1^ H_2_SO_4_ (see Section S4, Supporting Information). Finally, the deposited surfaces were placed in deionized water for 10 min to remove residual electrolytes. For the “sub‐add” surfaces, a pre‐etched layer was first prepared by transposing the two electrodes and applying a constant current density of 0.4 A cm^−2^ for 60 s as a sufficient operating condition, thus providing a rougher initial surface for the subsequent deposition steps. The pre‐etched surface was next placed in ethanol solution and washed in an ultrasonic bath for 15 min. Then the surface was placed in the cathode again and treated with the same deposition procedures to finally obtain the “sub‐add” surfaces. Besides, the “sub” surface through only etching was also fabricated as a comparison. The diagram of the formation process of the two kinds of surface and corresponding SEM images are shown in Figure 1. From Figure 1, it is evident that compared to “add” surfaces, “sub‐add” surfaces are characterized by larger dendrites, deeper holes, and larger average roughness. In addition, some void holes can be seen from the side view of SEM images on “sub‐add” surfaces. This is in agreement with the assumption as mentioned before.

### Pool Boiling Experiment

A schematic and photograph of the pool boiling setup are shown in Figure S4 (Supporting Information). The pool boiling experimental setup mainly consisted of a water chamber, auxiliary heating system, heating block, data acquisition system, and power supply. Details of the boiling test rig with schematics, experimental procedure, and measurement uncertainty are available in Sections S2 and S3 (Supporting Information).

### Quenching Boiling Experiment

A schematic and photograph of the pool boiling setup are shown in Figure S34 (Supporting Information). The quenching boiling experimental setup mainly consists of an electric actuator, a tube furnace, a quenching pool made of quartz glass, a heating platform, a high‐speed camera, and a data acquisition instrument. Details of the quenching boiling test rig with schematics, experimental procedure, and measurement uncertainty are available in Section S10 (Supporting Information) and the previous work.^[^ ^74^ ^]^

### Surface Wickability Measurements

The wickability measurement setup is shown in Figure S29 (Supporting Information). The wickability of surfaces was quantified using the liquid level drop method.^[^ ^18^ ^]^ Details of the experimental setup, calculation of wicking number Wi and Wi_Ra,_ and images of wicking tests are available in Section S8 (Supporting Information).

### Surface Characterization

The surface morphology was characterized by a SEM (Hitachi SU‐70) at an accelerating voltage of 3 kV and a 3D optical profiler (Veeco NT9100).

### Bubble Dynamics Statistics

The nucleation site density *n*_a_ was obtained by counting the average number of nucleation sites in at least five photos and dividing it by the surface area. The average bubble departure frequency *f* and average bubble departure diameter *d* were also obtained based on data from at least five nucleation sites. The generation time of each bubble was measured from the moment the bubble started to nucleate until it completely detached from the surface. The departure frequency was the reciprocal of the average departure time. The average bubble departure diameter was measured when the bubble just detaches from the surface.

## Conflict of Interest

The authors declare no conflict of interest.

## Supporting information



Supporting Information

Supplementary Video 1

Supplementary Video 2

Supplementary Video 3

Supplementary Video 4

Supplementary Video 5

Supplementary Video 6

Supplementary Video 7

## Data Availability

The data that support the findings of this study are available from the corresponding author upon reasonable request.
