# Supplementary material for: High‐Performance Boiling Surfaces Enabled by an Electrode‐Transpose All‐Electrochemical Strategy
Source: Adv Sci (Weinh). 2024 Dec 25;12(7):2413142. doi: 10.1002/advs.202413142 (PMC11831566; doi:10.1002/advs.202413142)
Supplement: Supplementary file 1 — Supporting Information [file ADVS-12-2413142-s002.docx]

**Supporting Information** for

High-Performance Boiling Surfaces Enabled by an Electrode-Transpose All-Electrochemical Strategy

Yu-Ming Chen^†^, Nan Hu^†^, Jia-Yi Zhang^†^, Yi-Fei Sun, Yue-Fei Wu, Zi-Rui Li, Li-Wu Fan^*^

Yu-Ming Chen, Yi-Fei Sun, Yue-Fei Wu, Zi-Rui Li

Institute of Thermal Science and Power Systems, School of Energy Engineering, Zhejiang University, Hangzhou 310027, China

Nan Hu

Department of Mechanical and Aerospace Engineering, Princeton University, Princeton, NJ 08544, USA

Jia-Yi Zhang

Department of Mechanical Science and Engineering, University of Illinois at Urbana-Champaign, Urbana, IL 61801, USA

^†^Y.M. Chen, N. Hu and J.Y. Zhang contributed equally to this work.

Li-Wu Fan

Institute of Thermal Science and Power Systems, School of Energy Engineering, Zhejiang University, Hangzhou 310027, China

State Key Laboratory of Clean Energy Utilization, Zhejiang University, Hangzhou 310027, China

^*^E-mail: [liwufan@zju.edu.cn](mailto:liwufan@zju.edu.cn) (L.W.Fan).

**S1 A brief review and comparative analysis of enhancing surface boiling heat transfer performance using electrodeposition and other fine-machining methods**

Figure S1 lists some surfaces oriented for better boiling heat transfer (BHT) performance constructed by precision-machining methods, and Figure S2 lists some surfaces enabled by electrochemical deposition (ECD) surfaces. Previous research suggests that surfaces with hierarchical structures, that is, surfaces with a micro-pillar array structure along with a micro-pit structure within the pillars, can enhance the surface's wicking capacity and provide abundant nucleation sites, thereby simultaneously improving critical heat flux (CHF) and heat transfer coefficient (HTC)^[1]^. On the other hand, the surfaces obtained from electrochemical treatment with their porous structure can aid in the enhancement of wickability, while the micro-dendrite structure on the skeletal structure of the pores can provide the surface with more nucleation sites. Such surfaces can meet the need for simultaneously improving the CHF and HTC. By manipulating several electrodeposition parameters, it is possible to obtain surfaces of diverse structures. These surfaces have also demonstrated good rates of improvement in boiling heat transfer performance (Table S1). Although the electro-deposition surface modification method has the advantages like easy to operate, low-cost, and easy to scale up, its boiling heat transfer performance is still somewhat inferior compared to surfaces obtained through other precision machining methods (Figure S3). The main reason is the weak controllability of the electrodeposition process, where excessive deposition at the base of the structure is inevitable during the process, thereby introducing unnecessary thermal resistance (Figure S3). In addition, due to structural strength limitations, there is a certain upper limit to the height of the dendrites at the top of the electro-deposited structure. Other fine-machining methods offer better controllability; geometric parameters such as the height of the structure, the spacing between micro-columns, etc., are controllable, and the base of the structure is unlikely to introduce unnecessary thermal resistance.


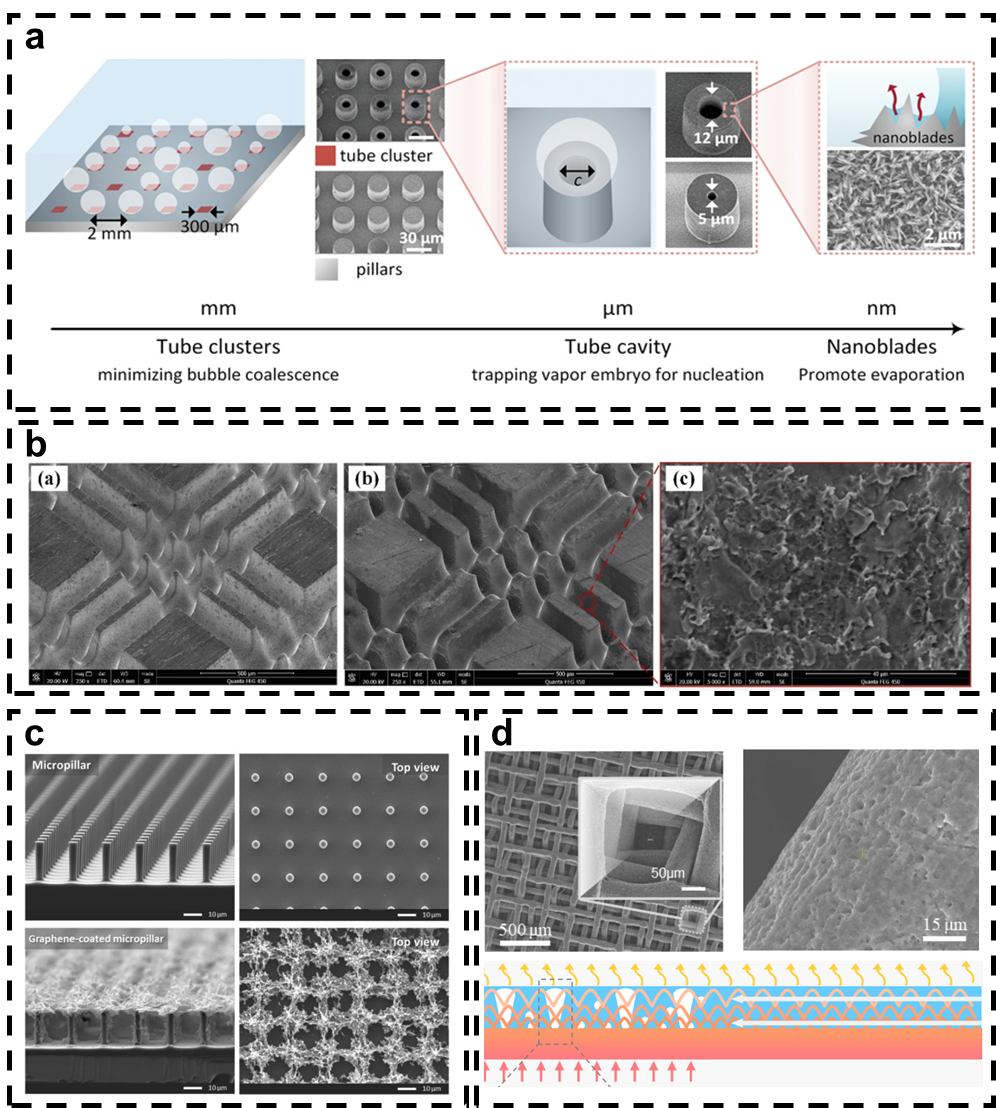


**Figure S1** Surfaces fabricated using fine-machining methods for enhancement of boiling heat transfer. a). Song et al.^[1]^ using lithography and deep reactive ion etching methods b). Tang et al.^[2]^ using electrical discharge machining method c). Choi et al.^[3]^ using MEMS (micro-electro-mechanical system) and rGO (reduced graphene oxide) nanofluid deposition d). Li et al.^[4]^ using chemical etching method and high-temperature sintering method of copper mesh. Pillar-like surfaces with hierarchical structures, characterized with superior wicking ability and abundant nucleate sites, can simultaneously enhance both HTC and CHF.


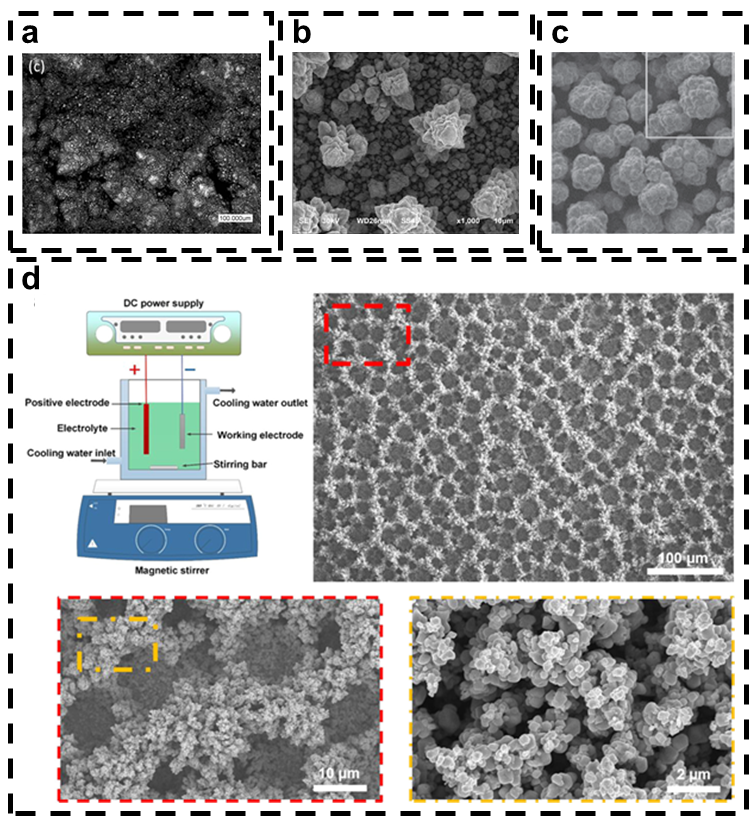


**Figure S2** Surfaces constructed by ECD method oriented for the enhancement of boiling heat transfer. a). Patil et al.^[5]^ b). Wang et al.^[6]^ c). Gheitaghy et al.^[7]^ d). Li et al.^[8]^ With porous and dendrites structures, surfaces enabled by ECD method can also be endowed with excellent wicking ability and abundant nucleate sites.

**Table S1** Summary of work related to enhancing pool boiling heat transfer by constructing structured surfaces using various strategies. (*x* M + *y* M represents an electrolyte with *x* mol/L CuSO_4_ and *y* mol/L H_2_SO_4_, electrolyte temperature was 25 ℃ unless specified).

| Author(s) | Electrochemical treatment process | CHF* | HTC* |
| --- | --- | --- | --- |
| **ECD-based bottom-up methods** | | | |
| Li et al.^[8]^ | Electrolyte: 0.2 M + 1 M, deposited at 3 A/cm^2^ for 30 s, then strengthened by high temperature sintering | 3.45 | 2.32 |
| Kalita et al.^[9]^ | Electrolyte: 0.8 M + 1.5 M, deposited on the CuO surface after chemical corrosion, deposited at 0.06 A/cm^2^ for 40 min | 1.71 | 1.78 |
| Hong et al.^[10]^ | Electrolyte: 0.4 M + 1.8 M, deposited at 1 A/cm^2^ for 1 min, then strengthened by high temperature sintering under N_2_ atmosphere | 1.67 | 3.69 |
| Gheitaghy et al.^[11]^ | Electrolyte: 0.4 M + 1.5 M, deposited at 0.6 A/cm^2^ for 30 s, then at 0.06 A/cm^2^ for 2500 s, deposited on U-shape microchannel | 2.10 | 3.90 |
| Rishi et al.^[12]^ | Electrolyte: 0.8 M + 1.5 M, deposited at 0.4 A/cm^2^ for 5 s, then at 0.04 A/cm^2^ for 2500 s, repeated three times in total. (6-step deposition) | 1.54 | 1.93 |
| Rishi et al.^[13]^ | Electrolyte: 0.8 M + 1.5 M + 2.0% mass fraction graphene nanoparticles (GNP), deposited at 0.4 A/cm^2^ for 15 s, then at 0.04 A/cm^2^ for 2500 s | 2.30 | 3.90 |
| Gupta et al.^[14]^ | Electrolyte: 200 g/L CuSO_4_⋅5H_2_O + 60 g/L H_2_SO_4_ + 10 g/L TiO_2_ particles, deposited at 0.3 A/cm^2^ for 50 s, then at 0.05 A/cm^2^ for 3000 s | 1.86 | 2.85 |
| Gupta et al.^[15]^ | Electrolyte: 200 g/L CuSO_4_⋅5H_2_O + 60 g/L H_2_SO_4_ + 10 g/L Al_2_O_3_ particles, deposited at 0.15 A/cm^2^ for 20 min | 1.73 | 3.73 |
| Mo et al.^[16]^ | Electrolyte: 0.6 M + 1.5 M, deposited at 1 A/cm^2^ for 60 s, a radial gradient distribution of electrolyte concentration enabled by injecting water at the center of the surface during electrochemical deposition process, then strengthened by high temperature sintering | 1.60 | 3.50 |
| Gheitaghy et al.^[17]^ | Electrolyte: 0.4 M + 1.5 M, deposited at 0.6 A/cm^2^ for 100 s then at 0.06 A/cm^2^ for 2500 s. electrolyte temperature was 60 ℃ | 1.50 | 3.70 |
| Gheitaghy et al.^[7]^ | Electrolyte: 0.4 M + 1.5 M, deposited at 0.25 A/cm^2^ for 50 s, then at a very low current for long time | 1.60 | 3.00 |
| Patil et al.^[5]^ | Electrolyte: 0.8 M + 1.5 M, deposited at 0.4 A/cm^2^ for 15 s, then at 0.04 A/cm^2^ for 2500 s | 1.43 | 3.75 |
| Wang et al.^[6]^ | Electrolyte: 0.6 M + 0.8 M, deposited at a linearly increasing current density from 0.1 A/cm^2^ to 0.3 A/cm^2^ for 300 s | 2.43 | 3.60 |
| Shakeri et al.^[18]^ | Electrolyte: 0.8 M + 1.5 M, deposited at 0.25 A/cm^2^ 50 s for twice, deposited on a “bi-conductive” surface enabled by wire electrical discharge machining (WEDM) process and epoxy coating | 1.62 | 3.60 |
| Shil et al.^[19]^ | First step: electrolyte: 0.8 M + 1.5 M + 2% mass fraction GNP, deposited at 0.4 A/cm^2^ for 15 s, then at 0.04 A/cm^2^ for 2500 s  Second step: electrolyte: mixture of 240 g/L CuSO_4_⋅5H_2_O, 40 g/L CuCl_2_⋅2H_2_O, 35 g/L H_3_BO_3,_ 0.2 g/L NaC_12_H_25_SO_4_ and 100 g/L Al_2_O_3_ nanoparticles, deposited at 0.06 A/cm^2^ for 1 h. | 2.22 | 3.39 |
| Pandey et al.^[20]^ | Electrolyte: 0.4 mol/L CuSO_4_ and 3 mol/L lactic acid, using NaOH solution to maintain the electrolyte at pH = 10, deposited at 2.0 V for 360 s. | 2.13 | 1.67 |
| **Top-down methods** | | | |
| Cooke and  Kandlikar^[21]^ | Computerized numerical control (CNC) milling | 1.95 | 3.74 |
| Zhong et al.^[22]^ | Face milling | 3.02 | 3 |
| Chu et al^[23]^ | Deep reactive ion etching (DRIE) | 2,6 | 1.99 |
| Gheitaghy et al.^[24]^ | Electrical discharge machining (EDM) | 1.65 | 2.7 |
| Song et al.^[25]^ | Lithography | 2.37 | 3.82 |
| A. Zou et al.^[26]^ | Deep ultraviolet (DUV) photolithography | 2.2 | 2.79 |
| **Other bottom-up methods** | | | |
| Xu et al^[27]^ | Self-assembly & Pyrolysis. N-doped carbon film | 1.84 | 2.98 |
| Zhou et al^[28]^ | Boiling thermal deposition. GO (graphene oxide) coating | 2.03 | 1.75 |
| Mao et al^[29]^ | Boiling thermal deposition. Carbon nanotubes (CNTs) porous coating | 1.86 | 1.73 |
| Das et al^[30]^ | Electron beam physical vapor deposition (PVD). Silicon oxide nanoparticle | 1 | 1.58 |
| Das et al^[31]^ | Electron beam PVD & annealing. TiO2 micro-nanostructures | 1 | 1.74 |
| Cheng et al^[32]^ | Method of boiling water treatment. Boehmite structure | 1.49 | 1.8 |
| Wang et al^[33]^ | Hydrothermal method Boehmite. structure | 2.62 | 2.81 |
| **Hybrid methods** | | | |
| Song et al^[1]^ | Pillar structure modified by using lithography and deep reactive ion etching methods | 2.38 | 4.89 |
| Tang et al^[2]^ | Two perpendicular electrical discharge machining processes | 2.29 | 3.62 |
| Choi et al^[3]^ | Micro-electro-mechanical system (MEMS) and reduced graphene oxide (rGO) nanofluid deposition | 2.52 | 3.88 |
| Li et al^[4]^ | High-temperature sintering method of copper mesh and chemical etching method | 3 | 3.32 |


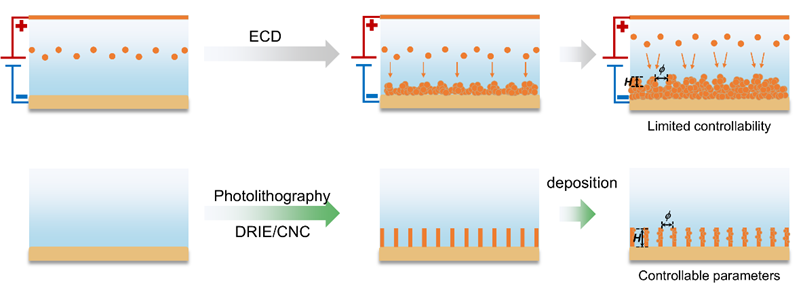


**Figure S3** Sketch for the relatively poor controllability of ECD methods compared with other fine-machining methods

**S2 Pool boiling experimental setup and experimental procedure**


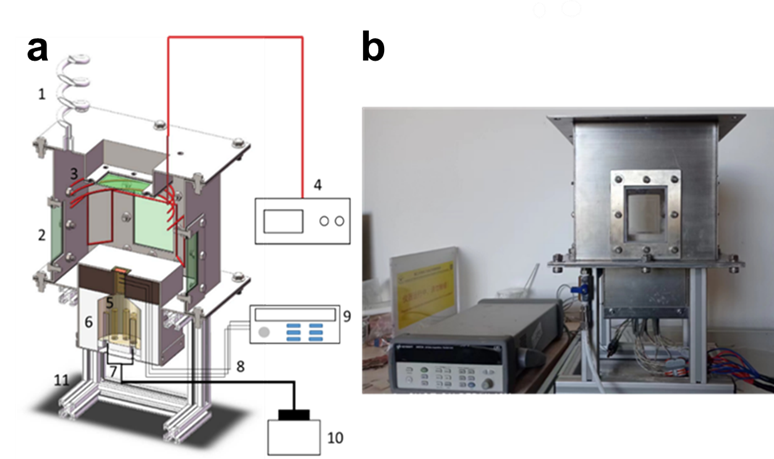


**Figure S4** a) schematic and b) photograph of the pool boiling experimental setup

1-condenser 2-water chamber 3,4-auxiliary heater 5-heating block 6-insulation layer

7-heating rods 8-T-type thermocouples 9-data acquisition system 10-transformer 11-holder

The pool boiling experimental setup mainly consisted of a water chamber, auxiliary heating system, heating block, data acquisition system (Agilent 34972A, Agilent Co. Ltd., USA), and power supply. The top area of the heating block was 20 mm × 20 mm. Three T-type thermocouples (WREK-191) were inserted into the heating block to collect the temperature of different heights of the heating block. Prior to each experiment, samples were first soldered onto the top of the heating block using a soldering tin (99.3%). Subsequently, the water chamber was filled with deionized water. Before testing, the water in the chamber was pre-heated by an auxiliary heater for about 2 h until the bulk temperature reached 100 ℃. Then, the water was continually degassed for 30 minutes. During the test, the transformer was initially adjusted to approximately 25 V, the data collection system was set to scanning mode, and the temperature data of three thermocouples was collected every 20 s. Every time a new heating voltage was adjusted, the temperature of the three thermocouples rose slowly until finally stabilized. When the temperature change within 1 min did not exceed 0.1 ℃, the input voltage could be increased. If the temperature of the thermocouples increased abruptly, the power supply was cut off immediately. For each test, roughly 20 data points were collected.

**S3 Data reduction, reliability and uncertainty analysis**

The heat flux and surface superheat are calculated based on the following equations:

|  |  | (S1) |  |
| --- | --- | --- | --- |
|  |  | (S2) |  |
|  |  | (S3) |  |
|  | |  | (S4) |

where *q’’* is the heat flux, *h* is the heat transfer coefficient, *k* is the thermal conductivity of copper, *T*_1_, *T*_2_ and *T*_3_ are the temperatures of three thermocouples from the top down. Δ*x* the distance between two adjacent thermocouples. Δ*x*_1_ is the distance between the top thermocouple and the top surface of copper block. And *δ* is the thickness of the samples. *T*_wall_ is the temperature of the top surface of sample. Since the thickness of soldering tin layer is very thin, its thermal resistance is neglected when calculating *T*_wall_.

According to the Rohsenow’s classic model^[34]^, for a smooth copper surface, with deionized water as the working fluid, the relationship between the heat flux and the surface superheat (that is, the surface temperature minus the saturation temperature of water) is:

|  |  | (S5) |
| --- | --- | --- |

According to Kandlikar's pool boiling heat transfer CHF prediction model^[35]^, for a smooth surface, the relationship between the CHF value during boiling and the surface receding contact angle *θ*_re_ is:

|  |  | (S6) |
| --- | --- | --- |

The symbols and parameters involved in the last two equations are defined as:

*θ*_re_: receding contact angle of surface and working fluid

*ρ*_v_: saturated vapor density, kg/m^2^

*ρ*_l_: saturated liquid density, kg/m^2^

Δ*H*_lv_: latent heat of evaporation, J/kg

*σ*: surface tension, N/m

*φ*: angle between test surface and horizontal plane, 0° in this work

*c*_p,l_: specific heat capacity of saturated water at constant pressure, J/(kg·K)

Δ*T*_sat_: wall superheat, K

*C*_sf_: empirical coefficient, for water-copper, 0.013.

*μ*_l_: dynamic viscosity of water, Pa·s

*k*_l_: thermal conductivity of water, W/(m·K)

*n*: empirical coefficient, for water, 1

To verify the reliability of the experimental setup, three consecutive boiling tests of the bare copper surface were performed and compared with the last two models. The results, depicted in Figure S5, demonstrate excellent repeatability of the three tests and great compatibility between the curves and these models. Additionally, the CHF value was compared with values from other works on bare surfaces. The CHF value in this work falls within a reasonable range, further proving the reliability of the experimental setup.

The primary sources of uncertainty in this work stem from the measurement error of the thermocouples and the error in thermocouple positioning. The uncertainties are ±0.1°C and ±0.4 mm respectively. The calculated uncertainty is about 26% when the heat flux is about 100 kW/m^2^, and the uncertainty is 5.6% when the heat flow density reaches 1000 kW/m^2^. At low heat flux, the temperature difference between the three thermocouples is small, leading to a large relative error in measuring the temperature interval. However, the measurement accuracy is within a reasonable range at higher heat flux, and the repeatability of experiments on the same surface is also acceptable.


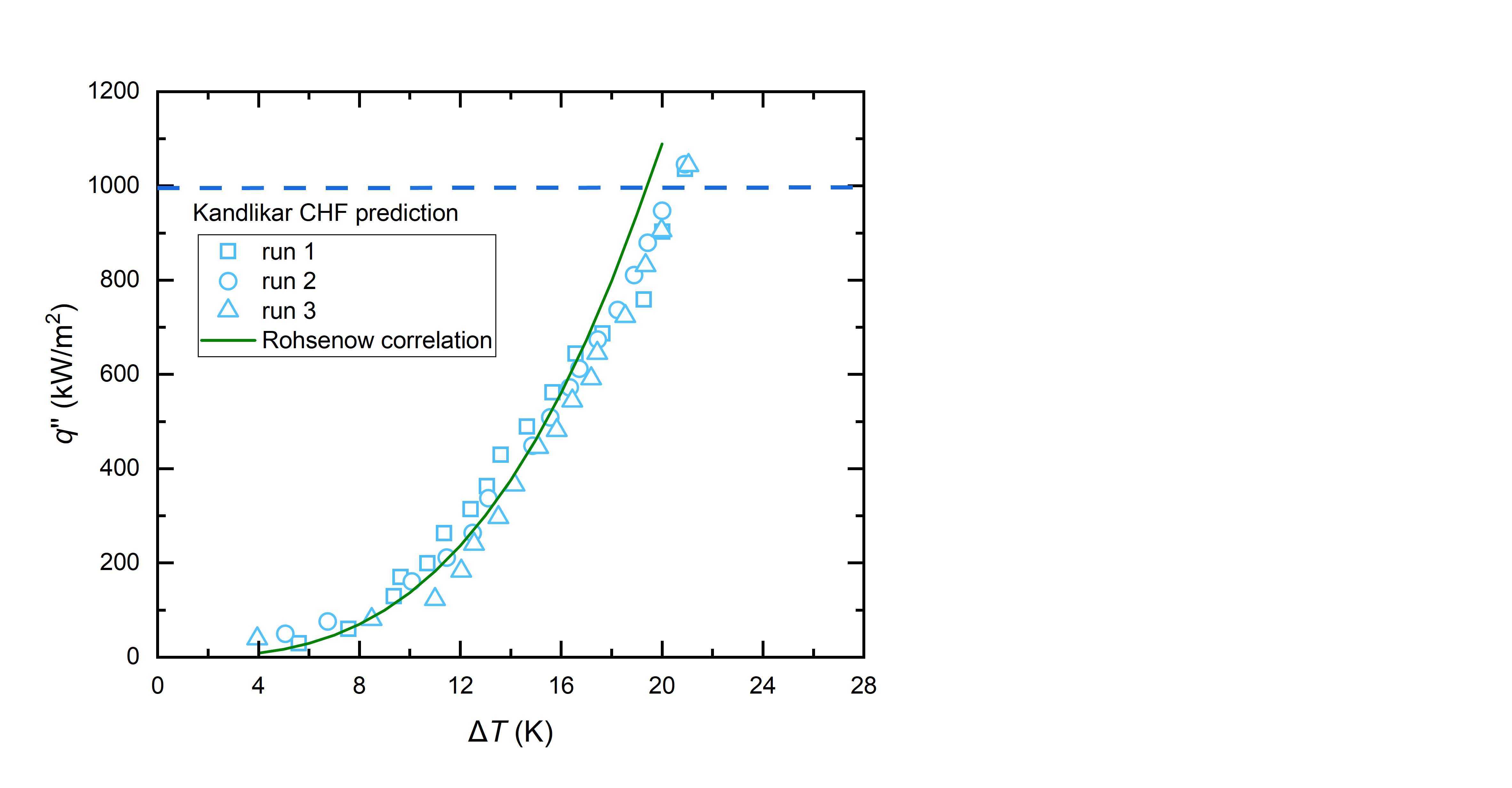


**Figure S5** The pool boiling heat transfer curve on the unmodified smooth copper surface is compared with two classic boiling models^[34][35]^.

**S4 Optimization of the electrochemical deposition procedure for “add” surfaces**

In this work, a 6-step electrochemical deposition procedure was applied to create porous structures that demonstrate superior pool boiling performance compared to surfaces made by the two-step electrochemical deposition method. The deposition procedure is shown in Figure S6. For each surface, a large current density was first applied to form the primary structure for a short time. Then, a very low current was applied for a much longer time to enhance the structure's robustness. These two steps were repeated two more times. In this work, *I*_0_ was fixed at 0.02 A/cm^2^ and the deposition time was 2500 s for every step unless specified.


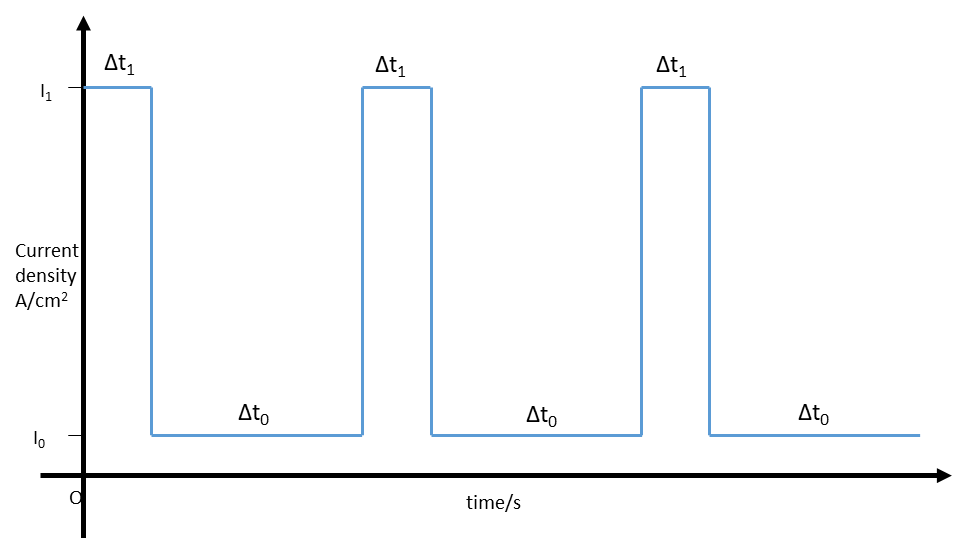


**Figure S6** 6-step electrochemical deposition procedure

Previous research has shown that for 2-step-deposited surfaces, current density (*I*_1_), deposition time (Δt_1_), electrolyte composition, temperature and electrolyte additives have significant impacts on surface morphology and boiling heat transfer (BHT) performance. Besides, 6-step-deposited surfaces exhibit better BHT performance compared to 2-step-deposited ones. However, to the best of the author’s knowledge, there has been no research focusing on the influence of *I*_1_, Δt_1_ and electrolyte composition on 6-step-deposited surfaces. Therefore, to discover the optimized deposition parameters for creating surfaces with superior BHT performance, a series of experiments were conducted. The BHT curves, SEM images and bubble photographs from these experiments are presented in this section. In the following discussion, for all the surfaces, the concentration of H_2_SO_4_ was fixed at 1 mol/L and the deposition temperature was 25 ℃.

**S4-1 Number of steps**

**Table S2** surfaces deposited by different number of steps. (The total electrodeposit time for each surface is fixed)

| Surface | Deposition procedure |
| --- | --- |
| 2-step | 0.4 A/cm^2^ for 90 s，0.02 A/cm^2^ for 7500 s |
| 4-step | 0.4 A/cm^2^ for 45 s，0.02 A/cm^2^ for 3750 s，repeated twice |
| 6-step | 0.4 A/cm^2^ for 30 s，0.02 A/cm^2^ for 2500 s，repeated three times |
| 8-step | 0.4 A/cm^2^ for 22.5 s，0.02 A/cm^2^ for 1750 s，repeated four times |


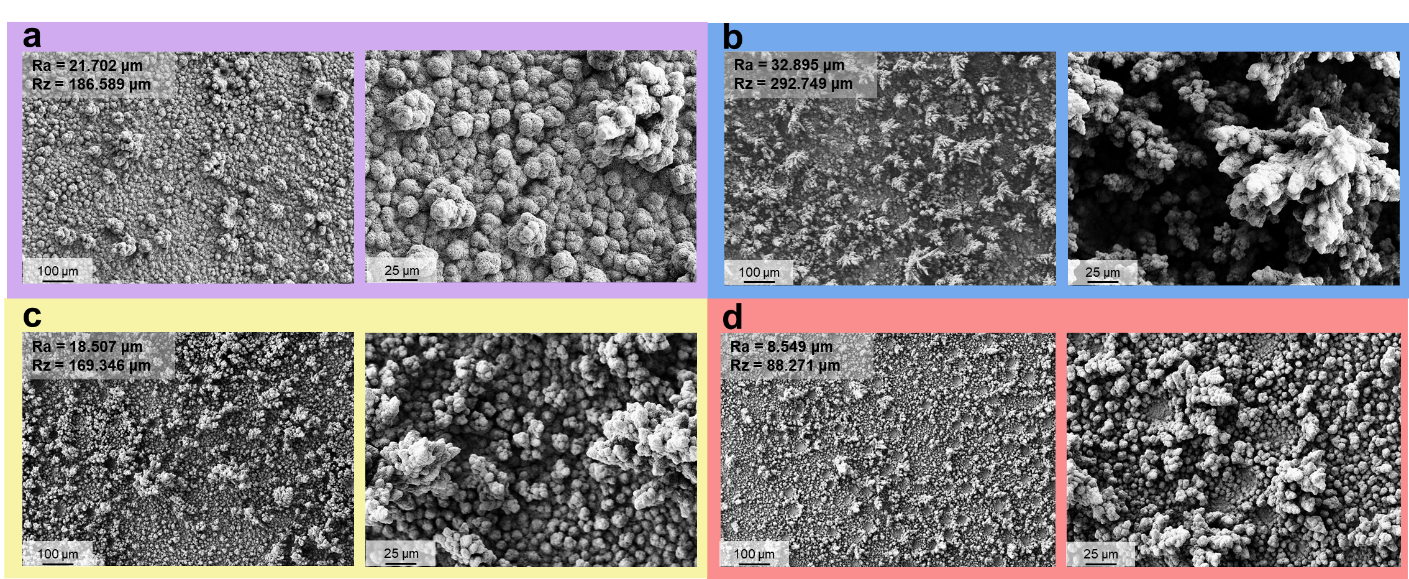


**Figure S7** SEM images of (a) 8-step (b) 6-step (c) 4-step and (d) 2-step surfaces show that with an increase in deposition steps, the number of dendrites protruding from the surface significantly increase, as does the height of the dendrites. The average roughness of the surface also significantly increases. However, the surface roughness decreases when the number of steps increases from six to eight.


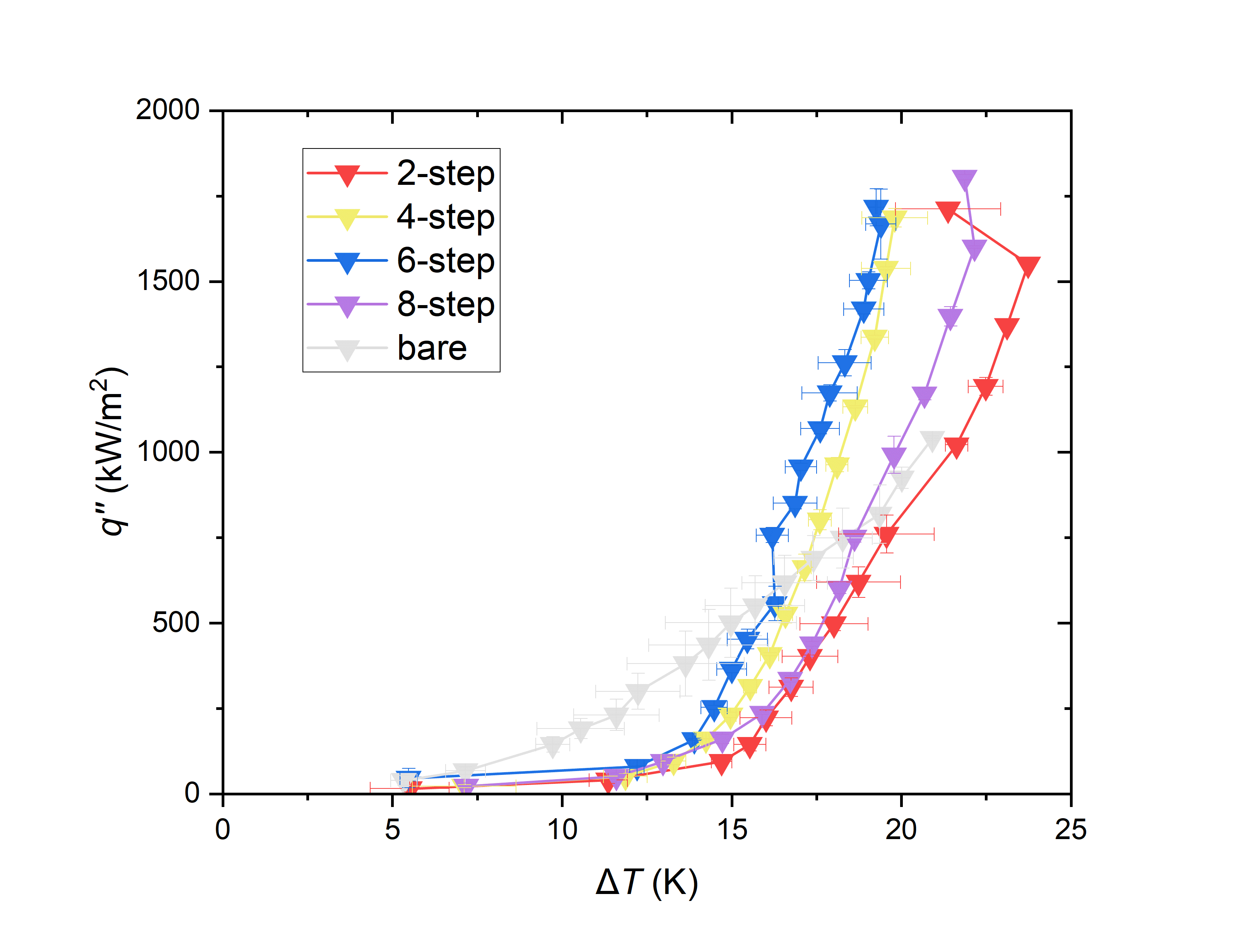


**Figure S8** Boiling curves of surfaces deposited by different steps reveal that the CHF values of all four surfaces reached approximately 1700 kW/m^2^, about 1.7 times that of untreated smooth copper plates. The CHF values of the four surfaces are close and show no obvious differences. However, the overall boiling curves clearly shows that the boiling curve of the 6-step surface is at the left, the 4-step and 8-step surfaces are in the middle, while the boiling curve of the 2-step surface is significantly to the right. At the same level of superheat, the heat flux of the surfaces is ranked as follows: 6-step > 4-step > 8-step > 2-step. This indicates that the 6-step surface has the highest heat exchange efficiency, and the HTC of the surface at each level of superheat is higher than the other surfaces. Under these experimental conditions, the 6-step surface displayed the best boiling heat transfer performance, so the subsequent studies in this section all adopt the six-step electrodeposition method.


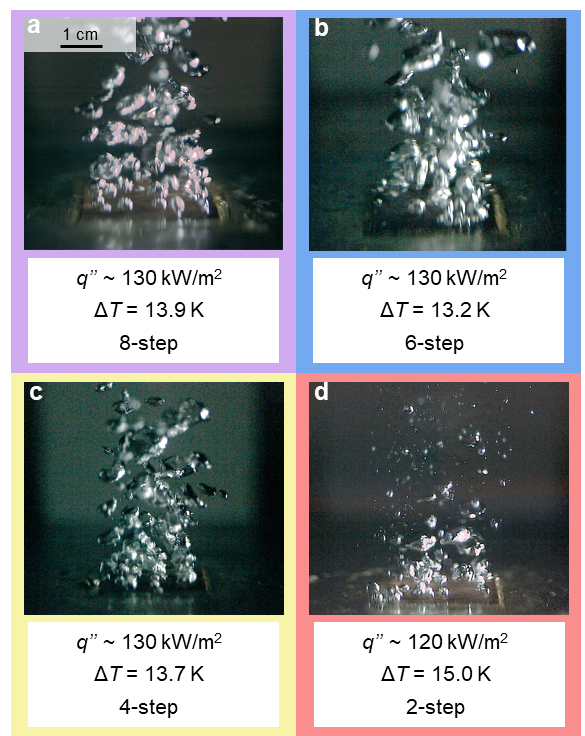


**Figure S9** Bubble images for (a) 8-step (b) 6-step (c) 4-step and (d) 2-step surfaces at low heat flux show distinct differences. The 2-step surface has fewer bubbles, a lower density of nucleation points, and greater superheat. In contrast, the other three surfaces have significantly more bubbles; at lower superheat, more nucleate sites are activated, and the 6-step electrodeposit surface has the lowest superheat. It can be seen that the surface obtained by more electrodeposition steps becomes rougher. This gives the surface with more nucleate sites, thereby increasing the HTC at this stage. However, when there are too many deposition steps, the surface roughness decreases instead, which is not conducive to the nucleation of bubbles at the low heat flux density stage, leading to a decrease in surface HTC.


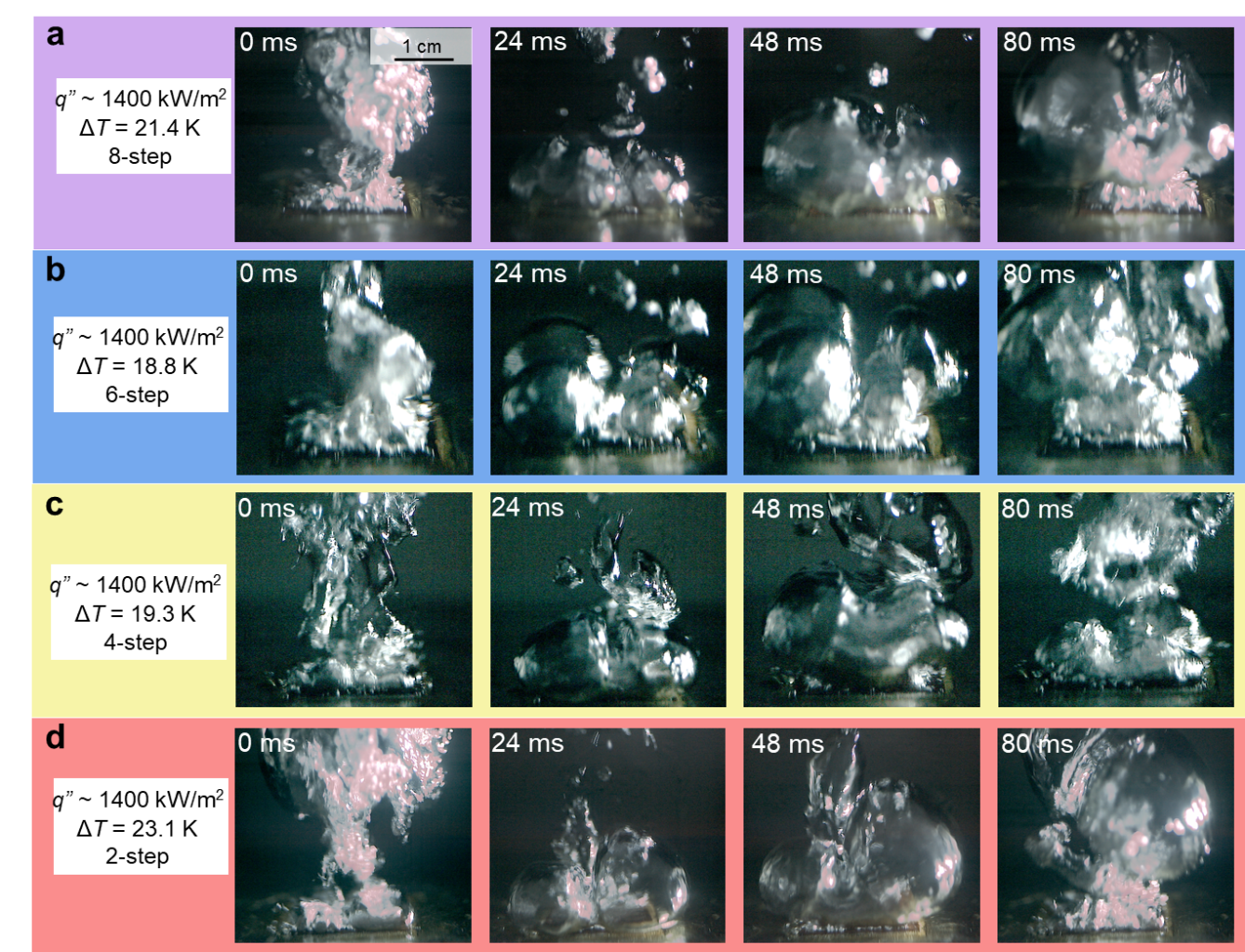


**Figure S10** Bubble images for (a) 8-step (b) 6-step (c) 4-step and (d) 2-step surfaces at high heat flux show that the bubble behaviors of the four surfaces are quite similar. Each boiling surface is covered with large bubble clusters that slowly detached from the surface, with the average detachment time for a group of bubble clusters is around 80 ms. The similarity in bubble behaviors among the four surfaces under high heat flux density also indicates that the boiling heat transfer performance at this stage is similar, with comparable CHF values.

**S4-2 Current density (*I*_1_)**

**Table S3** Surfaces deposited by different *I*_1_ (electrolyte and deposition time are fixed)

| surface | CuSO_4_ | Current density | Deposition time |
| --- | --- | --- | --- |
| 1 | 0.2 mol/L | 0.4 A/cm^2^ | 30 s |
| 2 | 0.2 mol/L | 0.65 A/cm^2^ | 30 s |
| 3 | 0.2 mol/L | 1 A/cm^2^ | 30 s |
| 4 | 0.2 mol/L | 1.5 A/cm^2^ | 30 s |


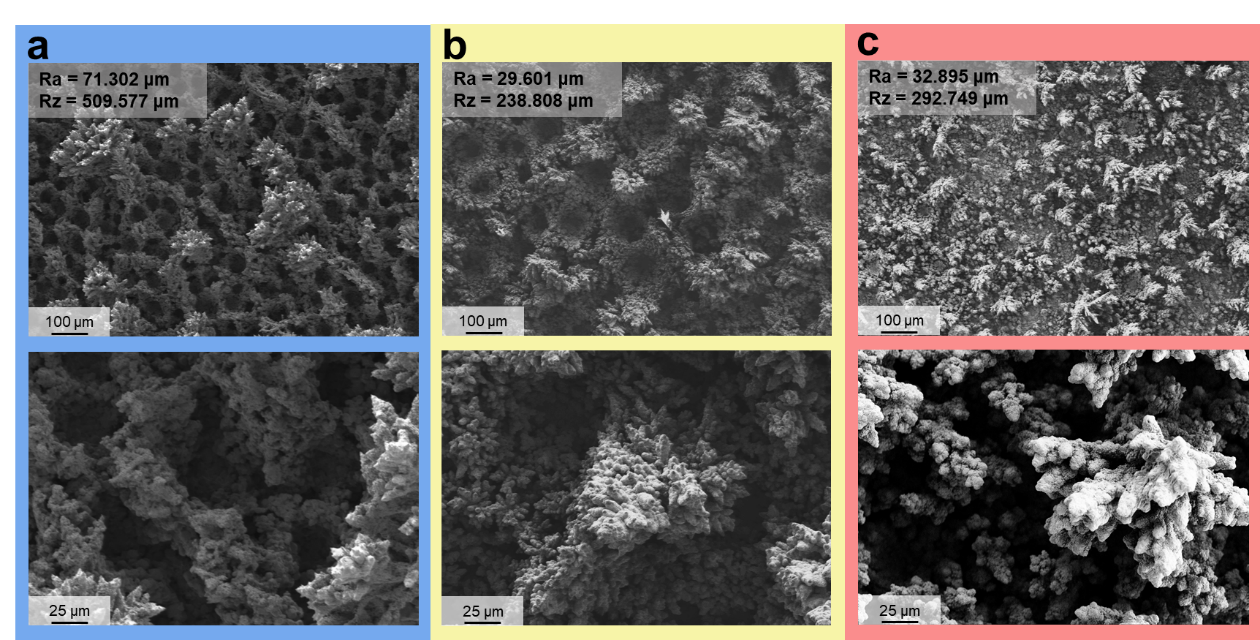


**Figure S11** SEM images of (a) surface 3, (b) surface 2, and (c) surface 1 show that as the current density increases, the structure of the surface gradually transitions from a dendritic array structure to a hierarchical porous structure.


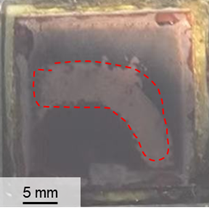


**Figure S12** Structural detachment occurred on Surface 4 during the boiling experiment. When the current density is too high, the stability of the surface structure decreases, making it unable to meet the requirements of the boiling experiment.


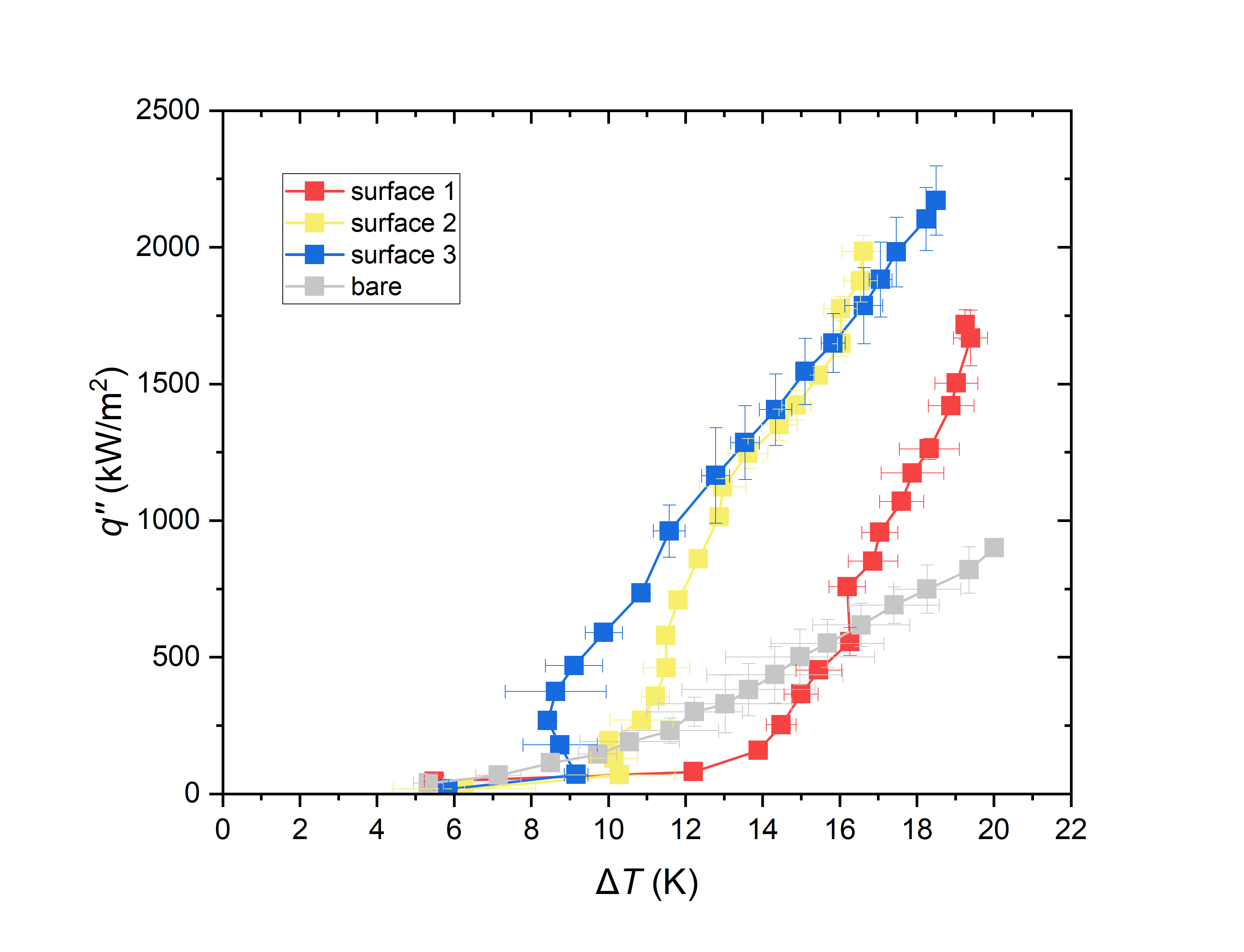


**Figure S13** Boiling curves of surfaces deposited by different *I*_1_ show distinct differences in heat flux density. For Surface 1, when ΔT < 14 K, the heat flux density of the surface rises slowly, remaining much lower than that of the untreated copper plate surface. Subsequently, the surface heat flux density rises rapidly, with the final CHF value reaching 1717 kW/m^2^, which is 91% higher compared to the untreated surface. For Surfaces 2 and 3, their overall boiling curves significantly shift to the left compared to Surface 1, demonstrating superior boiling heat transfer performance. The heat flux densities of Surfaces 2 and 3 begin to rise rapidly at ΔT ~ 9 K and ΔT ~ 10 K, respectively, and then steadily increase. Ultimately, the CHF value of Surface 2 reaches 1984 kW/m^2^, and Surface 3 reaches 2170 kW/m^2^, representing increases of 91% and 109% compared to the untreated copper plate, respectively.


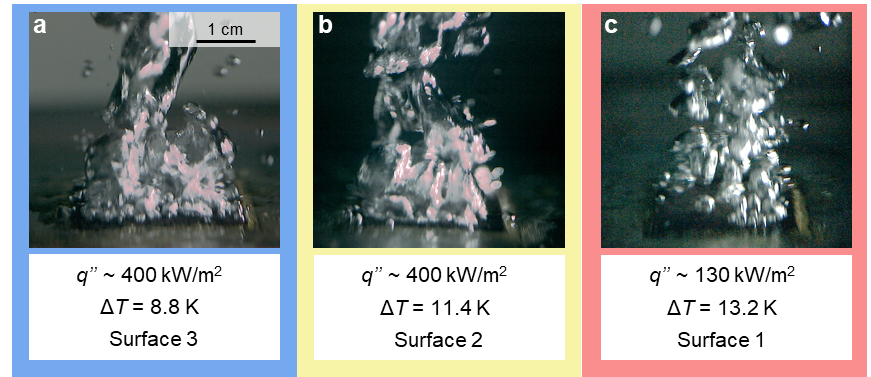


**Figure S14** Bubble images for(a) surface 3, (b) surface 2, and (c) surface 1 at low heat flux. Compared to Surface 1, bubbles generate at almost all locations on Surface 2 at a lower degree of superheat. The number of activated nucleate sites significantly increases, while a large part of Surface 1 still shows no bubble generation. This indicates that the porous micro-pit structure of Surface 2 provides abundant nucleation sites, enabling more bubbles to be generated at a lower degree of superheat, thereby significantly enhancing the HTC value of the surface. Compared to Surface 2, Surface 3 exhibits an even lower degree of superheat at the same heat flux density, further increasing the HTC value at this stage.


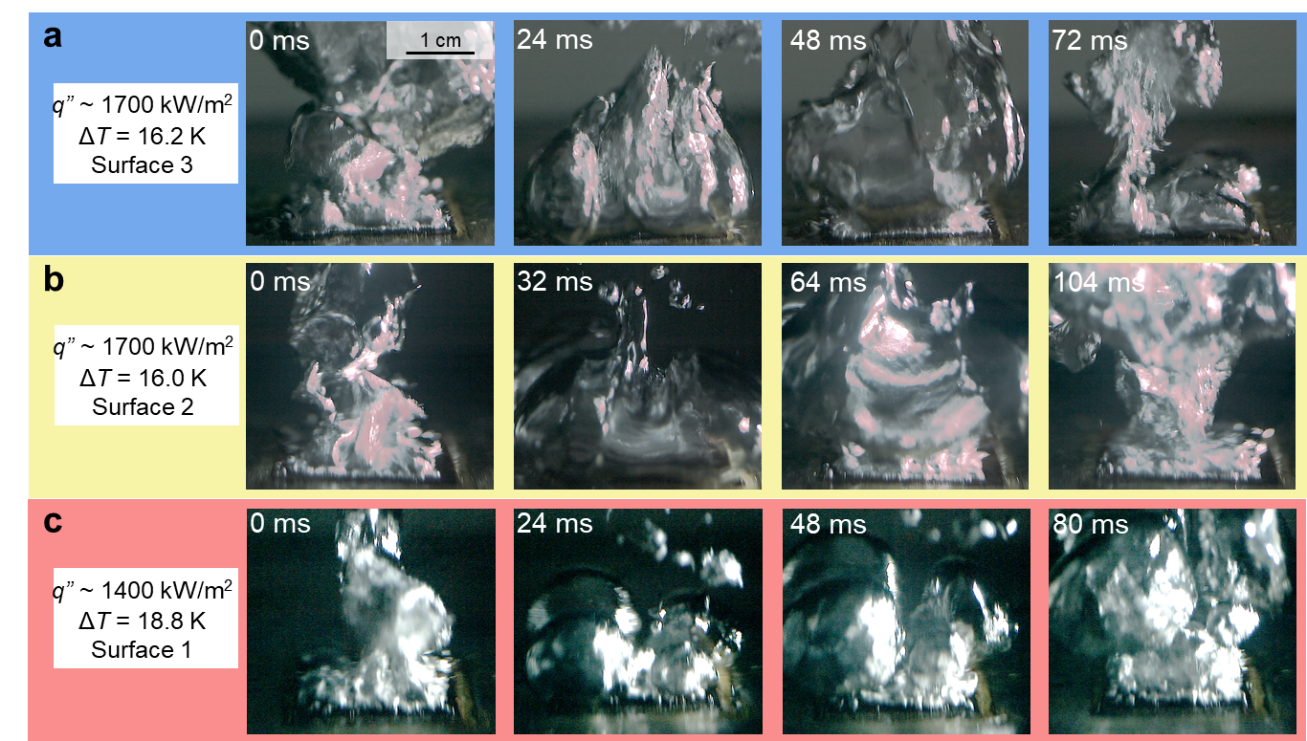


**Figure S15** Bubble images for (a) surface 3, (b) surface 2, and (c) surface 1 at high heat flux reveal important insights into bubble behavior. The bubble behavior of Surface 3 at a heat flux density of about 1700 kW/m^2^ is quite similar to that of Surface 1 at 1400 kW/m^2^. Bubbles on both surfaces intensively merge into groups and detach from the boiling surface. The overall size of the bubble groups and the detachment time are similar. At the same heat flux, the size of the bubble groups on Surface 2 is significantly larger than that of Surface 1, and the detaching time of the bubble groups is longer. Comparing the bubble behaviors at this stage, Surface 3 demonstrates the best heat transfer performance at a high heat flux density stage.

**S4-3 Deposition time (Δ*t*_1_)**

**Table S4** Surfaces deposited by different Δ*t*_1_(Electrolyte and current density are fixed. Since surface 3 in S4-2 with a current density of 1 A/cm^2^ showed best result, current density in this part is fixed at 1 A/cm^2^)

| surface | CuSO_4_ | Current density | Deposition time |
| --- | --- | --- | --- |
| 3 | 0.2 mol/L | 1 A/cm^2^ | 30 s |
| 5 | 0.2 mol/L | 1 A/cm^2^ | 20 s |
| 6 | 0.2 mol/L | 1 A/cm^2^ | 10 s |
| 7 | 0.2 mol/L | 1 A/cm^2^ | 40 s |


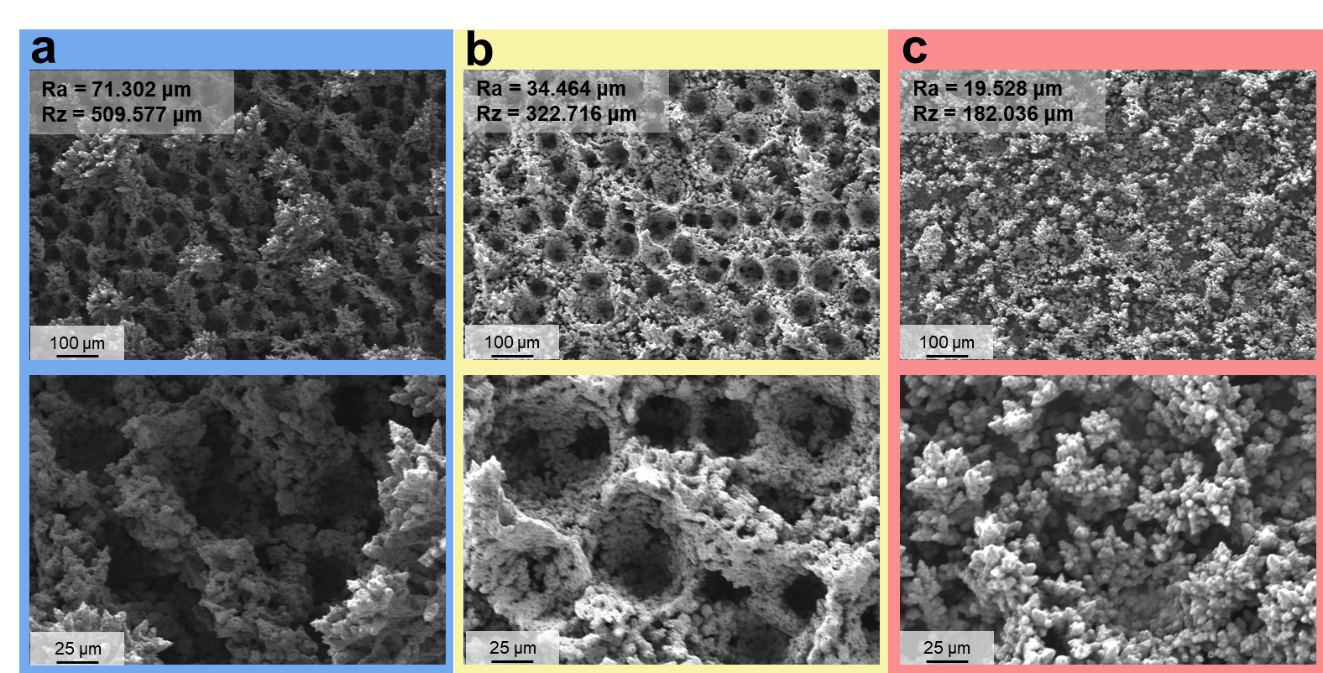


**Figure S16** SEM images of (a) surface 3, (b) surface 5, and (c) surface 6 reveal distinct differences in surface morphology. Surface 6 is lined with more short dendrites, without showing an obvious pore structure. When the deposition time increases to 20 seconds, the morphology of Surface 5 becomes similar to that of Surface 3, exhibiting a porous structure. The overall pore size of Surface 5 is slightly larger than Surface 3, and the pore structure is relatively orderly. In contrast, Surface 3 has some longer dendritic protrusions above the overall pore structure. Additionally, with the increase in deposition time, the roughness of the surface shows an upward trend.


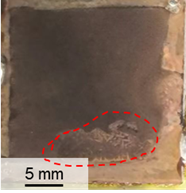


**Figure S17** Structural detachment occurred on Surface 7 during the boiling experiment. When the deposition time is too long, the stability of the surface structure decreases, making it unable to meet the requirements of the boiling experiment.


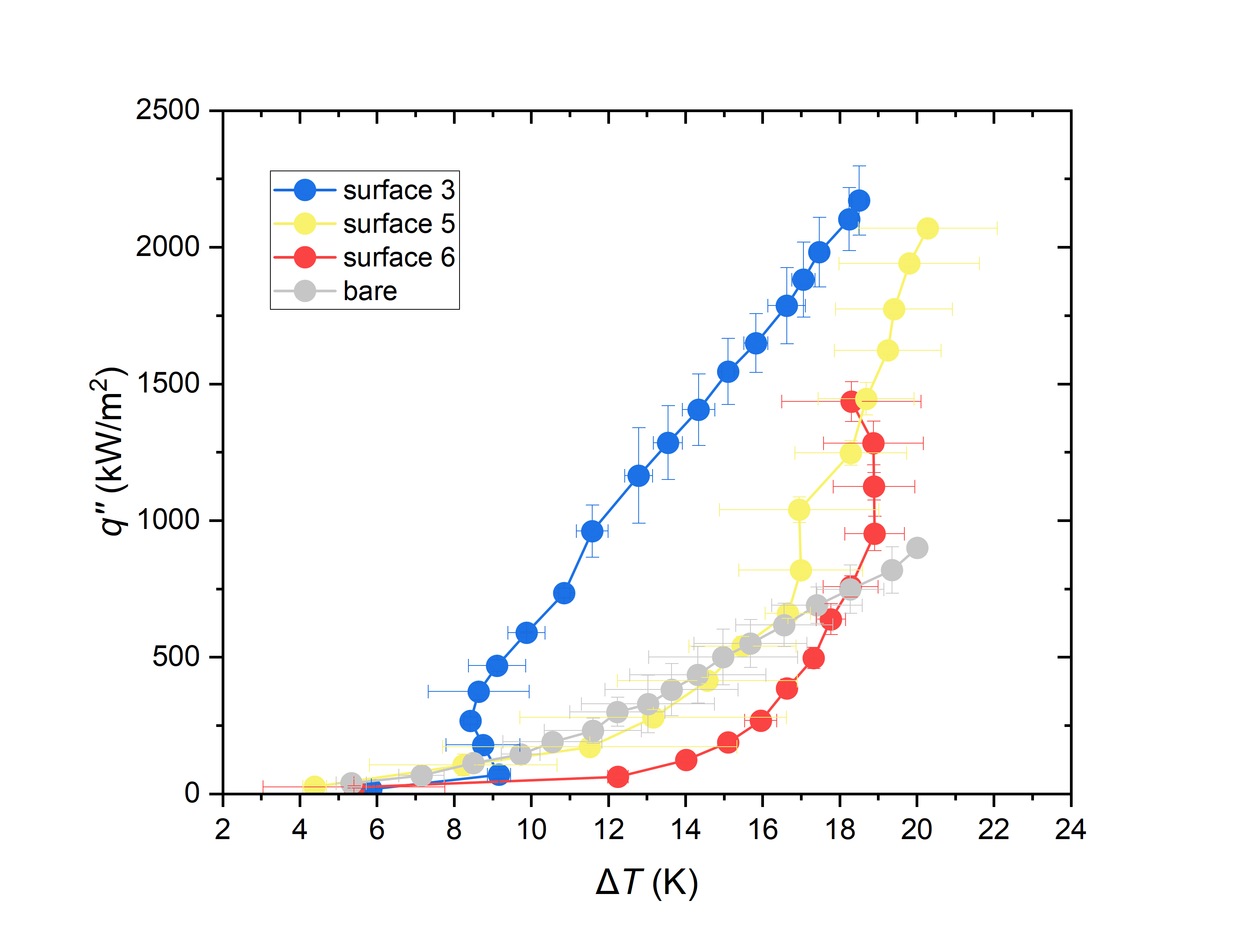


**Figure S18** Boiling curves of surfaces deposited by different Δ*t*_1_ show varying heat transfer performance. For Surface 6, the heat transfer performance is poor when Δ*T* < 18 K, with the boiling curve below that of the untreated copper plate. After that, the surface heat flux density rapidly rises, ultimately reaching a CHF of 1435 kW/m^2^, a 38% increase compared to the untreated surface. The boiling curve of Surface 5 is close to that of the untreated plate when Δ*T* < 16 K. Subsequently, the surface heat flux density rapidly increases, reaching its CHF at about Δ*T* ~ 20 K. The CHF value is 2069 kW/m^2^, a 99% increase compared to the untreated copper plate. Surface 5's CHF value is close to that of Surface 3, but the overall boiling curve is to the right of Surface 3. Accordingly, it can be seen that under a certain deposited current density, increasing deposition time is beneficial for enhancing surface boiling heat transfer performance.


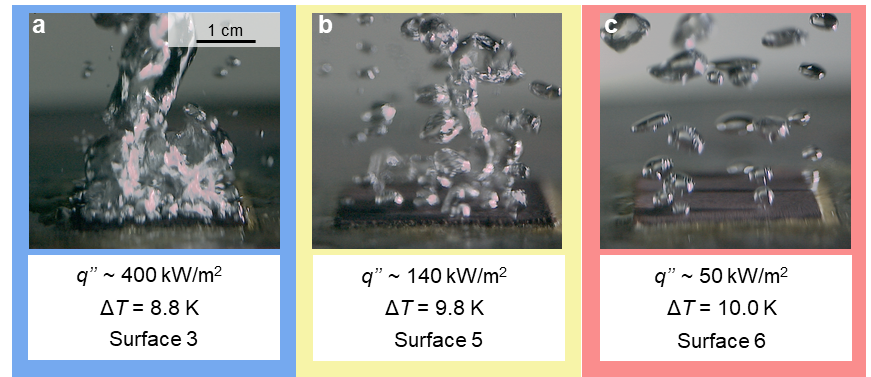


**Figure S19** Bubble images for (a) surface 3, (b) surface 5, and (c) surface 6 at low heat flux show distinct differences in boiling performance. Surface 6 performs poorly at low heat flux density, with only a few activated nucleation sites even when the surface superheat reaches 10 K. The majority of the surface area remains in the region of natural convection. The boiling heat transfer performance of Surface 5 is slightly improved, with multiple activated sites on the surface at a similar level of superheat, but there are still some areas where bubbles are not generated. Compared to these two surfaces, Surface 3 has abundant activated nucleate sites at a superheat of about 8.8 K, generating a large number of bubbles. It demonstrates significantly better boiling heat transfer performance than the other two surfaces, and the HTC value of Surface 3 has been greatly improved.


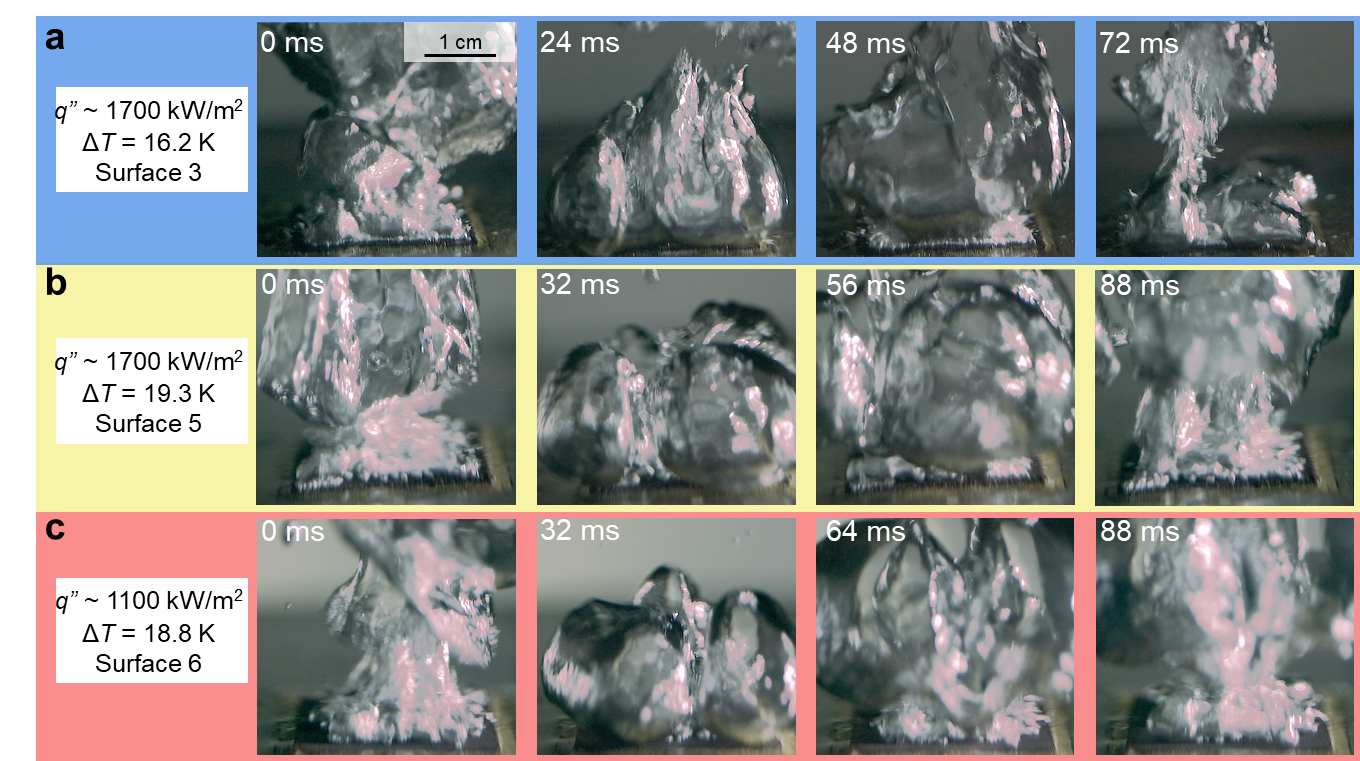


**Figure S20** Bubble images for (a) surface 3, (b) surface 5, and (c) surface 6 at high heat flux. Surface 6 exhibits large bubble clusters slowly detaching from the surface when the heat flux density is approximately 1100 kW/m², whereas Surface 5 demonstrates a similar bubble behavior to Surface 6 when the heat flux density increases to 1700 kW/m^2^. The size of the bubble clusters and detachment frequency in both cases are roughly equivalent. Compared to Surface 5, Surface 3 has a lower surface overheat at the same heat flux density and a slightly higher bubble cluster detachment frequency, indicating a faster rewetting rate for this surface. Consequently, the boiling heat transfer performance of Surface 3 is the best at this stage of heat flux density, and this surface also has the highest CHF value.

**S4-4 Concentration of CuSO_4_**

**Table S5** Surfaces deposited by different concentration of CuSO_4_ (since surface 9 was fragile, surface 10 with deposition time of 15 s was introduced)

| surface | CuSO_4_ | Current density | Deposition time |
| --- | --- | --- | --- |
| 3 | 0.2 mol/L | 1 A/cm^2^ | 30 s |
| 8 | 0.1 mol/L | 1 A/cm^2^ | 30 s |
| 9 | 0.4 mol/L | 1 A/cm^2^ | 30 s |
| 10 | 0.4 mol/L | 1 A/cm^2^ | 15 s |


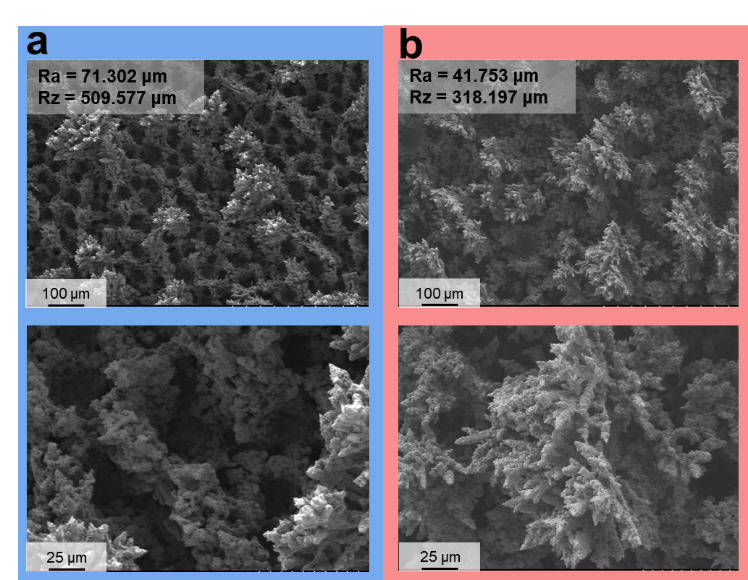


**Figure S21** SEM images of (a) surface 3 and (b) surface 10 reveal notable differences. Compared to Surface 3, Surface 10 also has a distribution of dendritic structures. However, unlike the porous structure at the bottom of Surface 3, the roughness of Surface 10 decreases, and there are virtually no clear pore structures at the bottom. Instead, it is completely covered by copper deposition particles. This deposition layer acts as an additional thermal resistance at the bottom of the surface. The heat flow inside the structure is more obstructed in the vertical direction, making it more difficult to activate the vaporization cores on top of the structure. This increased obstruction could, in turn, increase the superheating required for bubble nucleation, significantly reducing the HTC value of the surface in the low heat flow region.

**Figure S22** Structural detachment occurred on Surface 8 and Surface 9 during the boiling experiment. Forming a stable deposition structure is challenging if the concentration of CuSO_4_ in the electrolyte is either too high or too low.


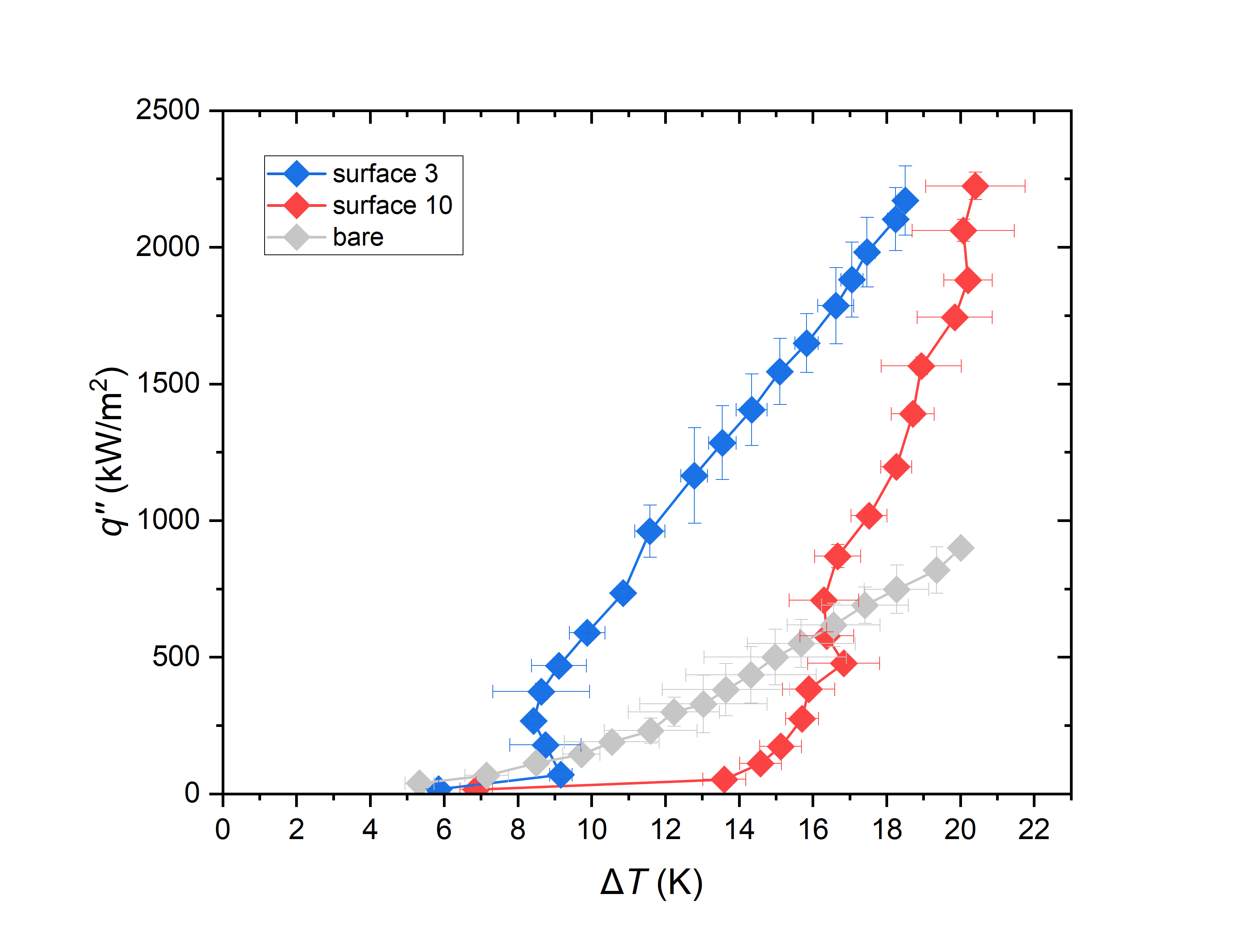


**Figure S23** Boiling curves of surfaces deposited with different concentrations of CuSO_4_ reveal significant differences in performance. For Surface 10, although the CHF value has reached 2224 kW/m^2^, an increase of 114% compared to the untreated copper plate, slightly higher than Surface 3, its overall boiling curve has noticeably shifted to the right. Surface 10's heat exchange efficiency in the medium and low heat flux density region is not satisfactory. Moreover, when Δ*T* is between 6 K and 14 K, there is almost no significant increase in the heat flux density of Surface 10.


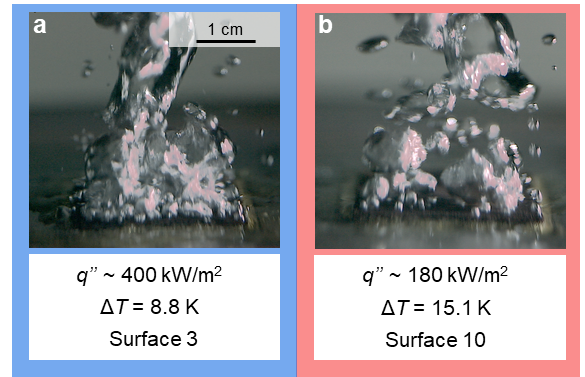


**Figure S24** Bubble images for (a) surface 3 and (b) surface 10 at low heat flux reveal important insights Under a superheat of 15.1 K, Surface 10 still only has sporadic spots where bubbles form. The majority of nucleation sites on the surface remain inactivated, leading to a very low HTC value at this stage, even much lower than that of the untreated copper plate surface. Meanwhile, for Surface 3, at a superheat of 8.8 K, almost all nucleation sites are activated, generating a large number of bubbles. When the CuSO_4_ concentration in the electrolyte is too high, the additional thermal resistance added to the surface's bottom layer significantly increases the superheat needed for bubble nucleation during the initial boiling stage, resulting in a noticeable deterioration of boiling heat transfer performance.


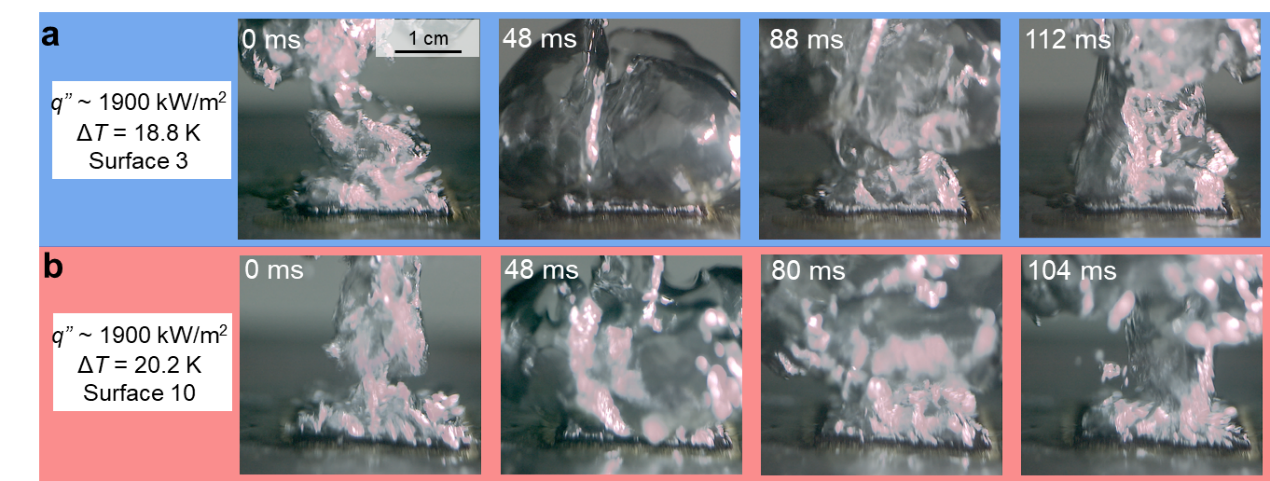


**Figure S25** Bubble images for (a) surface 3 and (b) surface 10 at high heat flux reveal key differences in bubble behavior. At this stage, compared to Surface 3, a few strands of bubble columns can still be discerned within the bubble clusters on Surface 10, and the detachment frequency of these bubbles is slightly faster. This suggests that at high heat flux density stages, Surface 10 has better wicking capability and a slightly higher rewetting rate than Surface 3, leading to a slight increase in the final CHF value for the surface.

**S4-5 Summary**

Based on the analysis in this section, under the conditions of this experiment, the boiling heat transfer performance of the surface can be improved by increasing the current density of the larger current steps or by increasing the deposition time. As the current density increases, the surface morphology transforms from a dendritic array structure to a hierarchical porous structure, and the surface roughness increases. Surfaces with a porous structure show a better boiling heat transfer performance at all heat flux density stages. Increasing the deposition time can make the porous structure of the surface clearer and increase its roughness, which is also beneficial for improving surface boiling heat transfer performance.

However, the increase in current density and deposition time cannot be infinite; there is a specific limit. Excessive deposition current density and deposition time will lead to a fragile deposited surface structure that cannot withstand a complete boiling heat transfer test. Additionally, when the H_2_SO_4_ concentration in the electrolyte is fixed, the CuSO_4_ concentration in the electrolyte should be moderate. A too-low concentration will cause the generation reaction of hydrogen bubbles to dominate in the deposition process, resulting in unstable surface deposition structures. Conversely, a too-high concentration will cause the deposition of copper particles to dominate excessively, increasing the deposition thickness at the structure's bottom, introducing unnecessary thermal resistance, and thus deteriorating the boiling heat transfer performance of the surface at the initial boiling stage.

**S5 BHT performance comparison of the “sub-add”, “add” surfaces with other works**

Given the evidence in Figure S26, it indicates that a better performance does not have to be achieved by more precise control of the micro and nanostructures, as seen in lots of grey scatters by ECD are superior to brown or green scatters. Especially, the hybrid method of MEMS+rGO deposition also introduces the random structures at submicron scale. The key point to achieve better boiling performance lies in the formation of multi-tiered structures in either ordered or disordered manner. Despite electrochemical methods not being able to precisely control single ion in deposition or removal, there are many pathways to sufficiently regulate structures in ECD methods including step number, current density, electrolyte and adding other particles etc. All these works cannot be seen as precisely controllable fabrication methods, while also can reach an impressive result, as shown as grey scatters in Figure S26.

The innovation of our work is the realization of the hybrid processing method using a full electrochemical process, i.e., top-down etching followed by bottom-up multi-step deposition. It avoids complex and costly processes such as photolithography, requires only electrode transposition in the same electrolyte, and adapts to curved surfaces. Although the geometry is not precisely uniform, it exhibits a statistically significant multi-tier structure and an impressive BHT performance in experiments.

The obtained “sub-add” surface based on the electrode-transpose strategy holds the best performance HTC and second rank in CHF comparing to conventional ECD methods, which makes a competitive comprehensive ability in thermal management with fine-machining method. As emphasized in the manuscript, this simple and ingenious strategy enables a low-cost, scalable and geometry-adaptive treatment of metal surface for engineering applications.





**Figure S26** BHT performance of “sub-add” surface and best “add” surface in S4 and comparison with other works utilizing ECD and other surface modification methods^[1]-[33]^. The boiling heat transfer performance of surfaces constructed through a simple, efficient, and low-cost electrodeposition method is equivalent to that of some surfaces constructed using more complex and costly precision machining techniques. (Abbreviations: GNP: graphenenanoparticles; GO: graphene oxide; CNT: carbon nanotubes; PVD: physical vapor deposition; CNC: computerized numerical control; DRIE: deep reactive ion etching; EDM: electrical discharge machining; DUV: deep ultraviolet; WEDM: wire electrical discharge machining; EDM: electrical discharge machining; MEMS: micro-electro-mechanical system; rGO: reduced graphene oxide.)

Table S6 shows the surface structure parameters and corresponding CHF and HTC enhancement ratio in some representative works. It can be seen that our “sub-add” surface had the largest surface roughness and dendrite length, and a relatively large pore size among all ECD surfaces. These features are all enabled by the innovative electrode-transpose strategy.

However, it should also be noted that, for ECD methods, not all the related work provided complete information about the surface structures. As listed in Table S6, some key features are missing more or less in these works, and the complexity of surface structure varies among these works; it is tricky to directly compare different available works and generate a correlation between BHT performance and surface geometrical parameters. For example, though we list the roughness, dendrite length, and pore diameter of these porous surfaces fabricated by electrochemical deposition methods in Table S6, it still lacks universal criteria to comprehensively characterize structures for quantitative analyses on the boiling heat transfer enhancement. In addition, even with relatively complete geometrical parameters of each surface, some other surface properties such as wickability and wettability cannot be accurately predicted by using these geometrical parameters. Table S7 exhibits the surface wickability and corresponding CHF and HTC enhancement ratio from previous works. It can be seen that the wickability measurement methods vary in different works. Even for the same method, the characteristics of the micro-nanostructure in Table S6 cannot fully correspond to the wicking results (compared with Li et al.^[8]^).

**Table S6**. Summary of structure parameters and corresponding CHF and HTC enhancement ratio in some previous works.

| Author(s) | Surface | Ra/μm | | Dendrite length/μm | Pore diameter/μm | CHF* | HTC* |
| --- | --- | --- | --- | --- | --- | --- | --- |
| This work | Sub-add | 109.1 | | 525.2 | 73.5 | 2.54 | 4.31 |
| Li et al.^[8]^ | ECD: Electrolyte: 0.2 M + 1 M, deposited at 3 A/cm^2^ for 30 s, then strengthened by high temperature sintering | / | | 112 | ~70 | 3.45 | 2.32 |
| Kalita et al.^[9]^ | ECD: Electrolyte: 0.8 M + 1.5 M, deposited on the CuO surface after chemical corrosion, deposited at 0.06 A/cm^2^ for 40 min | 7.79 | | / | / | 1.71 | 1.78 |
| Hong et al.^[10]^ | ECD: Electrolyte: 0.4 M + 1.8 M, deposited at 1 A/cm^2^ for 1 min, then strengthened by high temperature sintering under N_2_ atmosphere | / | | / | 80 | 1.67 | 3.69 |
| Gupta et al.^[15]^ | ECD: Electrolyte: 200 g/L CuSO_4_⋅5H_2_O + 60 g/L H_2_SO_4_ + 10 g/L Al_2_O_3_ particles, deposited at 0.15 A/cm^2^ for 20 min | 1.16 | | / | 3.1 | 1.73 | 3.73 |
| Wang et al.^[6]^ | ECD: Electrolyte: 0.6 M + 0.8 M, deposited at a linearly increasing current density from 0.1 A/cm^2^ to 0.3 A/cm^2^ for 300 s | / | | 230 | / | 2.43 | 3.60 |
| Song et al^[1]^ | Pillar structure modified by using lithography and deep reactive ion etching methods | | 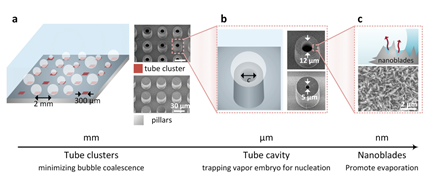  Pillar height: 30 μm, pillar distance: 2mm, pillar diameter: 300 μm, CHF*: 2.38, HTC*: 4.89 | | | | |
| Tang et al^[2]^ | Microgroove array surface modified by electrical discharge machining method | | 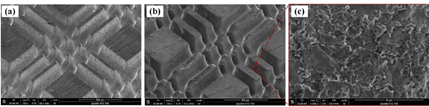  Pillar width: 400 μm, pillar height: ~100 μm, fin thickness: 20 μm, CHF*: 2.29, HTC*: 3.62 | | | | |
| Choi et al^[3]^ | Graphene oxide-coated micropillar surface using MEMS and rGO nanofluid deposition | | 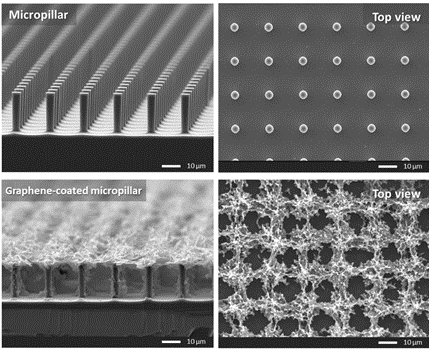  Pillar height: 20 μm, pillar pitch: 20 μm, pillar diameter: 4 μm, CHF*: 2.52, HTC*: 3.88 | | | | |

**Table S7**. Summary of surface wickability and corresponding CHF and HTC enhancement ratio in some previous works.

| Author(s) | Surface treatment process | Wickability measurement | Wi | V”  /(mm^3^/s) | U”  mm/s | CHF* | HTC* |
| --- | --- | --- | --- | --- | --- | --- | --- |
| This work | Sub-add | Liquid level drop method | 4.7 | / | 18.2 | 2.54 | 4.31 |
| Li et al.^[8]^ | ECD: Electrolyte: 0.2 M + 1 M, deposited at 3 A/cm^2^ for 30 s, then strengthened by high temperature sintering | Liquid level drop method | 12 | / | / | 3.45 | 2.32 |
| Kalita et al.^[9]^ | ECD: Electrolyte: 0.8 M + 1.5 M, deposited on the CuO surface after chemical corrosion, deposited at 0.06 A/cm^2^ for 40 min | / | / | / | / | 1.71 | 1.78 |
| Hong et al.^[10]^ | ECD: Electrolyte: 0.4 M + 1.8 M, deposited at 1 A/cm^2^ for 1 min, then strengthened by high temperature sintering under N_2_ atmosphere | Time for liquid absorption | / | / | / | 1.67 | 3.69 |
| Gheitaghy et al.^[7]^ | ECD: Electrolyte: 0.4 M + 1.5 M, deposited at 0.25 A/cm^2^ for 50 s, then at a very low current for long time | Liquid rise experiment | / | 4.38 | / | 1.60 | 3.00 |
| Gheitaghy et al.^[17]^ | ECD: Electrolyte: 0.4 M + 1.5 M, deposited at 0.6 A/cm^2^ for 100 s then at 0.06 A/cm^2^ for 2500 s. electrolyte temperature was 60 ℃ | Liquid rise experiment | / | 3.9 | / | 1.50 | 3.70 |
| Rishi et al.^[12]^ | ECD: Electrolyte: 0.8 M + 1.5 M, deposited at 0.4 A/cm^2^ for 5 s, then at 0.04 A/cm^2^ for 2500 s, repeated three times in total. (6-step deposition) | Droplet spread experiment | / | 0.01 | / | 1.54 | 1.93 |
| Pandey et al.^[20]^ | ECD: Electrolyte: 0.4 mol/L CuSO_4_ and 3 mol/L lactic acid, using NaOH solution to maintain the electrolyte at pH = 10, deposited at 2.0 V for 360 s. | Liquid level drop method | / | / | 30-50 | 2.13 | 1.67 |
| Song et al^[1]^ | Pillar structure modified by using lithography and deep reactive ion etching methods | / | / | / | / | 2.38 | 4.89 |
| Tang et al^[2]^ | Microgroove array surface modified by electrical discharge machining method | / | / | / | / | 2.29 | 3.62 |
| Choi et al^[3]^ | Graphene oxide-coated micropillar surface using MEMS and rGO nanofluid deposition | Droplet spread experiment | / | Wicking rate:  2.37 mm/s^0.5^ | | 2.52 | 3.88 |

**S6 Durability test and morphology change for the “sub-add” surface**

The morphological changes of surfaces before and after the durability tests are shown in Figure S27. The "sub-add" surface still maintains a relatively intact porous structure after the test, with several protruding dendrites on the porous structures, and the surface morphological characteristics are consistent with the original state prior to the test.


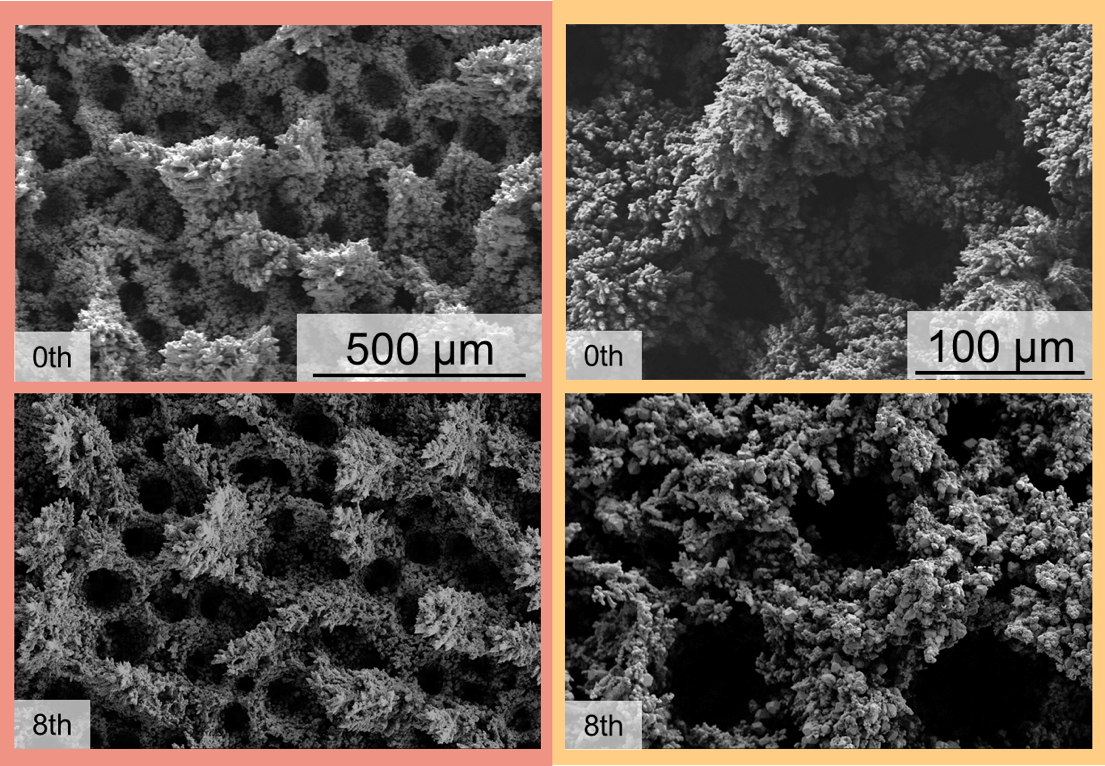


**Figure S27** SEM images for “sub-add” surface before and after durability tests. The top two images are SEM images for surfaces before tests and the bottom two images are for the surfaces after tests.

**S7 Mechanisms of nucleate boiling HTC enhancement at low heat fluxes**

In accordance with the classical heterogeneous nucleation theory^[34]^, following the bubble departure, the boiling surface enters a waiting period. There is no obvious bubble growth at this nucleation site during this period, but the temperature profile over the thermal boundary layer keeps developing on the wall with continuously increased superheat. Once the superheat of the liquid microlayer attains the critical temperature, rapid bubble growth is initiated. This growth is predominantly governed by the evaporation of the superheated liquid layer and affects the bubble departure dynamics. The schematic of the simplified force analyses on the bubble in the *y*-direction is depicted in Figure S28^[37]^. Here, the buoyancy force tends to lift the bubble away from the surface, while the surface tension force and the growth force work to retain the bubble on the surface.


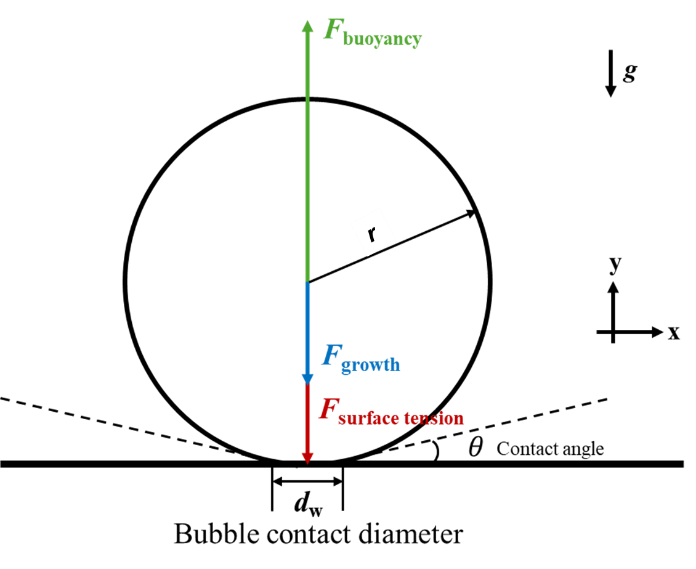


**Figure S28.** The simplified force balance model on a single vapor bubble attached to a horizontal boiling surface.

The equations for calculating these three forces are as follows^[37]^:

 (S7)

 (S8)

 (S9)

where *ρ*_l_, *ρ*_v_, *σ*, and *d*_w_ are liquid density, vapor density, surface tension and bubble contact diameter, respectively. *r*, $\dot{r}$, and $\ddot{r}$ are the bubble radius, the first derivative and the second derivative of the bubble radius, respectively, and *r* can be obtained from the model proposed by Zuber^[38]^:

 (S10)

 (S11)

where *α*_l_, *c*_p,l_, Δ*H*_lv_, and Δ*T*_sat_ are the liquid thermal diffusivity, specific heat, latent heat, and wall superheat, respectively. The bubble growth force^[39]^ represents the unsteady force exerted on a bubble when it grows on a surface during pool boiling. As a bubble is growing, the pressure inside the bubble is higher than the liquid pressure far from the bubble, and the pressure difference between the liquid-vapor interface and the liquid far from the bubble can be described using the Rayleigh equation:

 (S12)

where *P* represents the pressure. Assuming that the bubble is symmetric on an upward horizontal surface, the force acting on the liquid above the bubble due to the bubble growth can be calculated by Eq. S8. Accordingly, the reaction force acting on the bubble interface from the above liquid to the bubble has the same value, but its direction is downward. This force prohibits the tendency of bubble departure.

For the “sub-add” and “add” wicking surfaces, the surface tension force in the *y*-direction can be neglected due to the almost zero contact angle, thereby simplifying the force balance to a comparison between buoyance force and growth force. For a given bubble diameter on different surfaces, the buoyancy force acting on the bubbles is identical. The bubble growth force then becomes the primary factor in determining whether a bubble will depart at this specific diameter. When the bubble growth force equals the buoyancy force at this given diameter, the bubble is at the quasi-departure state and is going to depart. If the bubble growth force is smaller than the buoyancy force at this diameter, the bubble has already departed. Conversely, if the bubble growth force exceeds the buoyancy force, the bubble remains attached to the surface. On surfaces with a lower bubble growth rate, the bubble growth force is relatively smaller. Consequently, the diameter at which buoyancy and bubble growth forces are equal will be smaller than a surface with a higher bubble growth rate. Since the bubble departure diameter is defined as the diameter where the bubble growth force equals the buoyancy force, a slower bubble growth rate results in a smaller bubble departure diameter.

According to the nucleating model proposed by Hsu^[40] [40]^, surfaces with larger cavities require lower wall superheat to initiate nucleation^[41]^. Consequently, the superheat of the liquid layer is lower on surfaces with larger cavity sizes, leading to a slower bubble growth rate and a smaller growth force, facilitating bubble departure at a smaller diameter. Our experimental results are consistent with the above theory. The “sub-add” surface has a larger cavity size represented by larger pore size and surface roughness. Meanwhile, the “sub-add” surface exhibits the lowest wall superheat (7.2 K) among all four types of surfaces and a smaller bubble departure diameter as compared to that of the “add” surface when the heat flux was controlled at around 70 kW/m^2^. Similar analyses can also be extended to explain the bubble departure diameter on the bare and "sub" surfaces.

However, for a surface with intricate micro/nanostructures, there are still many other factors affecting both the bubble departure diameter and bubble departure frequency, such as the heat fluxes, wall superheats, and wettability^[42]^. Therefore, it is also not rigorous to attribute the faster bubble departure frequency and smaller departure diameter to the higher roughness and larger pore size without considering other potential effects, although many experimental results observed similar relationships^[43]^.

**S8 Wicking volume test on the “add” and “sub-add” surfaces**

To explain the difference in bubble behavior on the two deposition surfaces, surface wickability tests were performed. The diagram of surface wicking volume test is shown in Figure S29. Surface wickability is an important parameter to predict CHF values of superhydrophilic surfaces whose contact angle is close to 0°^[44]^. The wickability of surfaces was quantified using the liquid level drop method^[45]^. During the testing process, the capillary tube with an inner diameter of 0.5 mm which absorbed ≈1.5 μL of distilled water was vertically positioned on the top of the sample. The lab jack was used to elevate the sample up to contact the PFA tube, initiating the wetting process. The high-speed camera was used to record the transient liquid level drop in the capillary PFA tube at a frame rate of 500 frames per second during wetting process. When the surface contacts a pendant water drop meniscus protruding from a PFA (perfluoroalkoxy) tube, the liquid in the tube will be absorbed into the surface structures via capillarity, resulting in a continuous drop of liquid level in the PFA tube. The absorbed liquid volume V can be calculated by V=πD^2^∆h/4, where d is the inner diameter of PFA tube and ∆h is the drop height of the liquid level.


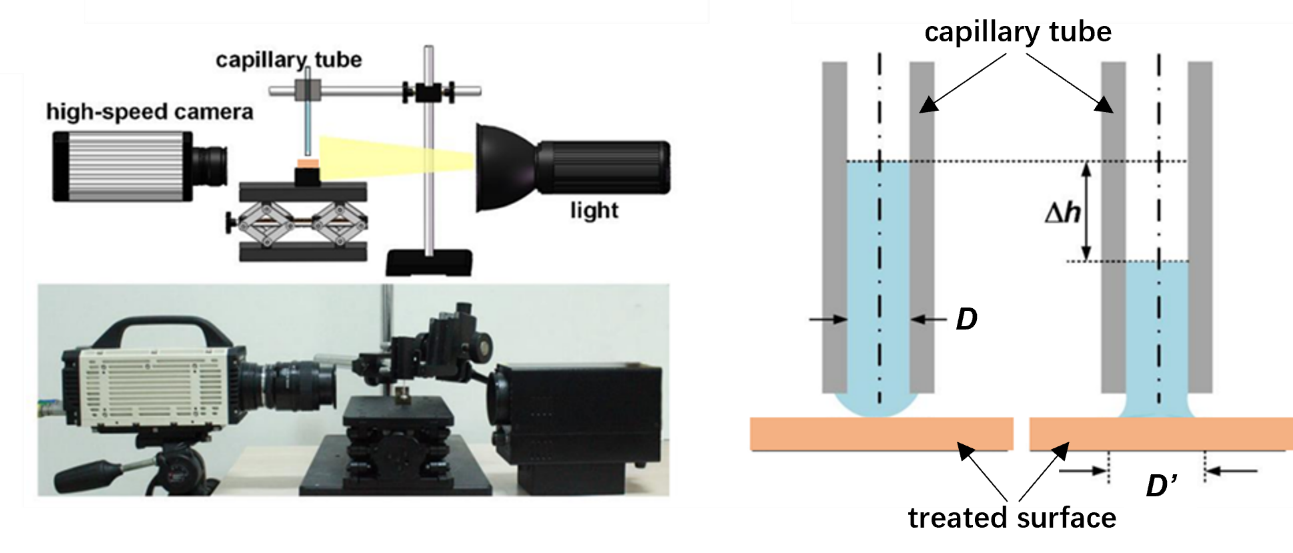


**Figure S29** Schematic diagram of the surface wicking volume test^[47]^

In current research on the influence of wickability on the boiling heat transfer process, most works adopt the method of analyzing the instantaneous liquid absorption rate *U*' at the beginning of the core absorption process^[46]^ (within about 20 ms):

|  |  | (S13) |
| --- | --- | --- |

The dimensionless wicking number Wi is characterized by the ratio of the instantaneous liquid absorption rate at the beginning of core absorption to the critical mass flow rate when steam leaves the surface during the boiling process^[44]^:

|  |  | (S14) |
| --- | --- | --- |

Zhang et al. pointed out that the enhancement effect of wickability on the boiling heat transfer process is also affected by surface roughness^[47]^. For surfaces with a larger roughness, the probability and frequency of bubble detachment and intermittent wetting during the boiling heat transfer process are greater. Therefore, the wicking process on the surface is more intense, and the enhancement caused by core absorption for boiling heat transfer is more obvious. Therefore, Zhang et.al proposed to weight the correction of the wicking number by the relative surface roughness factor *η*, with the expression for the corrected core absorption number Wi_Ra_ as follows^[47]^:

|  |  | (S15) |
| --- | --- | --- |

where Ra_wicking_ is the roughness of modified surfaces, Ra_unmodified_ is the roughness of unmodified surface, and *η* is the roughness ratio.

Figure S30 shows the changes in the liquid level in the PFA tube during the core absorption test for the "sub-add" surface and "add" surface in the 0-100 ms time period. In 0-20 ms, the liquid levels in the tube on the two deposited surfaces both dropped significantly, with no obvious increase in the drop height of the "sub-add" surface compared to the "add" surface.

It should be emphasized that the hole diameter *φ* is a statistic value for the holes perpendicular to the substrate, while the wickability test describing the main capability of water horizontal transport cannot be used to represent the hole diameter *φ*. Representative imbibition speed U ~ γ/μ (surface tension/viscosity) can be evaluated as ~50 m/s (~50 mN/m / 1 mPa s) for saturated water. From the side view of structure in Figure 1b and 1c, the heterogenous and multiscale nature of the porous structures can be easily observed. If we take ~100 μm as an effective pore size, the characteristic time of filling one pore is ~2 μs. Assuming 10^3^ to 10^4^ holes could consist of a representative element volume (REV) region, it implies the characteristic time for REV is ~2 ms to 20 ms. Hence, the heterogeneity may result in the overlap experimental results of wickability during the initial stage.

In the subsequent time period, the drop in the liquid level in the tube of "sub-add" surface was significantly greater than that of the "add" surface, and ultimately, the amount of liquid absorbed at 100 ms was significantly greater for the "sub-add" surface than for the "add" surface. Table S8 shows the result of *U’*, Wi, Wi_Ra_ for the “bare”, “add” and “sub-add” surfaces. The absorption rate and Wi of “sub-add” surface are slightly higher than those of the “add” surface. While considering surface roughness, Wi_Ra_ of the “sub-add” surface is much higher than that of the “add” surface.


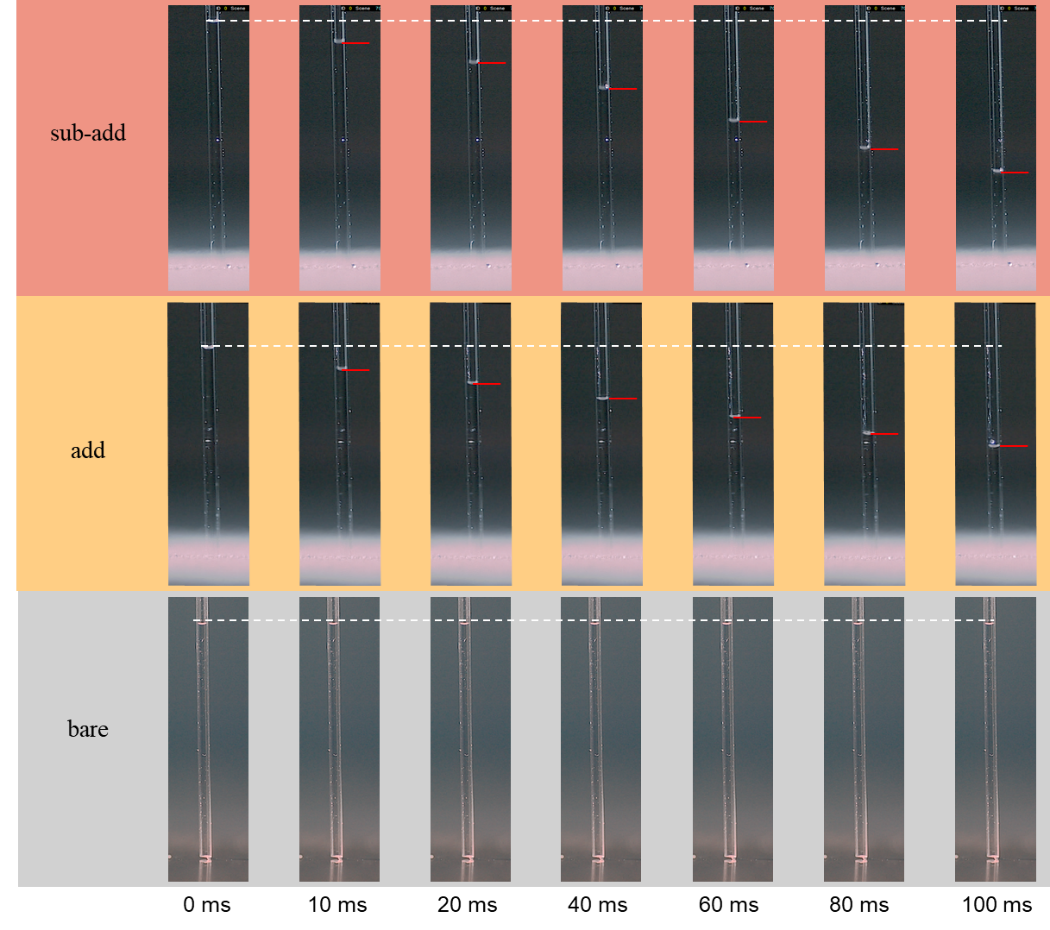


**Figure S30** images of liquid drop level tests for “sub-add”, “add” and “bare” surfaces

**Table S8** Results of absorption rate, Wi, Wi_Ra_ and corresponding CHF* for the “bare”, “add” and “sub-add” surfaces

| Surface | *U’* (μL/(mm^2^ s)) | Wi | Wi_Ra_ | CHF* |
| --- | --- | --- | --- | --- |
| bare | 0 | 0 | 0 | 1 |
| sub-add | 18.20±0.61 | 4.734±0.159 | 1885±63.31 | 2.539±0.015 |
| add | 17.83±0.18 | 4.550±0.046 | 1229±12.43 | 2.087±0.140 |

**S9 Bubble behaviors of the “add” and “sub-add” surfaces at different heat fluxes**

Consecutive snapshots of bubble evolution on the “add” and “sub-add” surfaces are given in Figure S31, S32, and S33. At a heat flux of around 1300 kW/m^2^ and 1900 kW/m^2^, the bubble columns on the “add” surface tended to coalesce horizontally and be stuck on the top of the surface, forming a blanket-like bubble cluster onto the surface. In contrast, the “sub-add” surface exhibited a different bubble behavior. Although horizontal coalescence still occurred, the coalesced bubble cluster had higher upward momentum, resulting in a column-like bubble cluster. In addition, the comparison of bubble evolution sequences between the “add” and “sub-add” surfaces also clearly implies that bubble departure frequency on the “sub-add” surface was higher than that on the “add” surface.


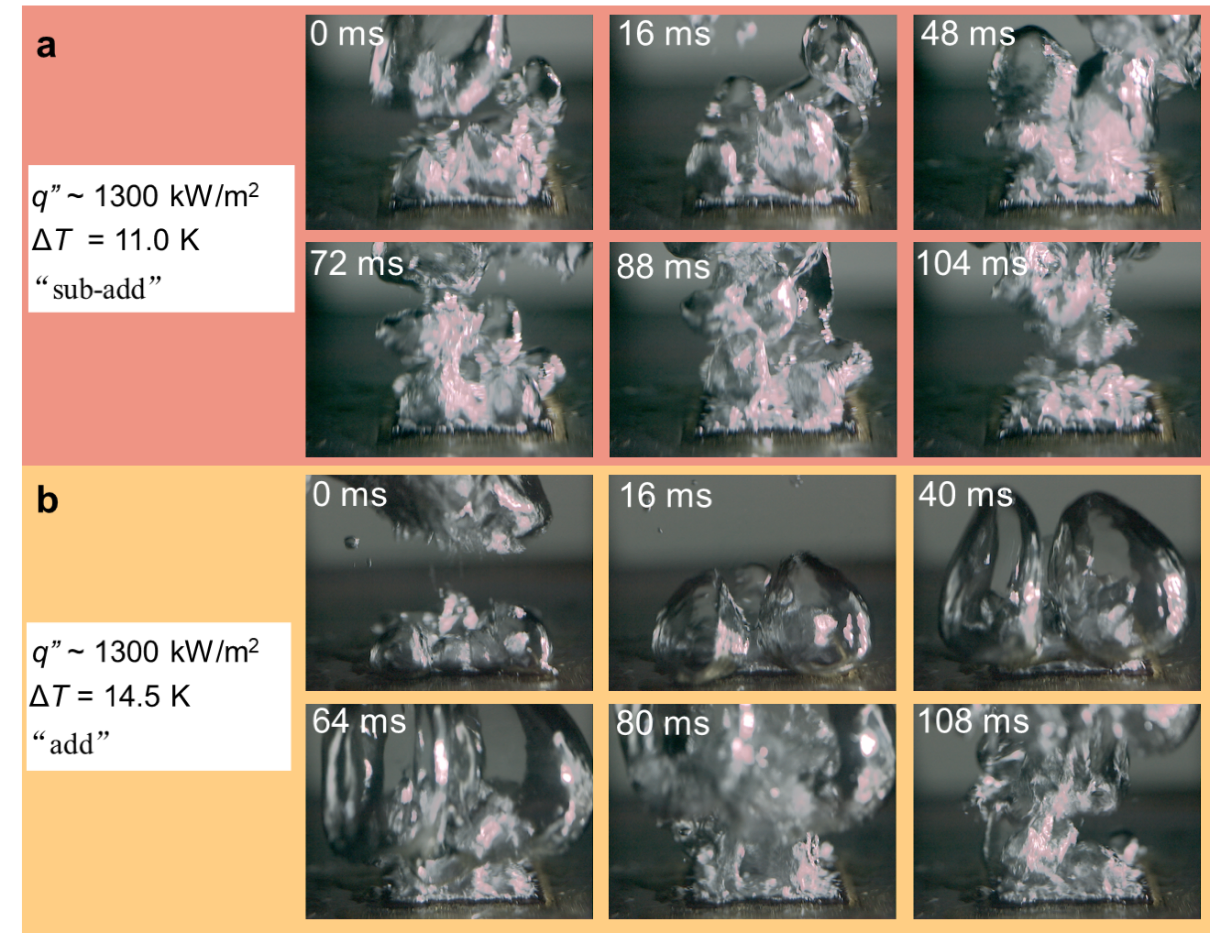


**Figure S31** Bubble images on the (a) “sub-add” surface and (b) “add” surface at the heat flux of *q''*~1300 kW/m^2^


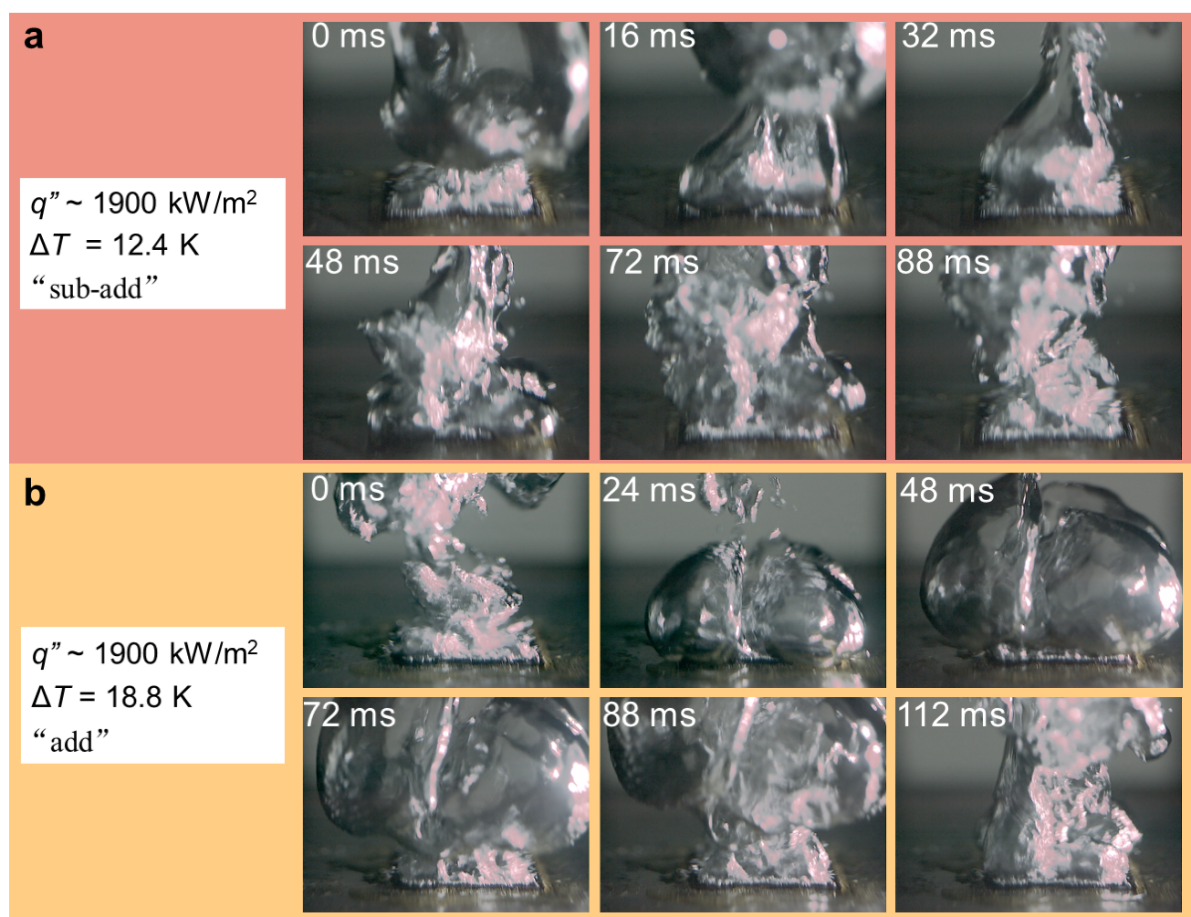


**Figure S32** Bubble images for the (a) “sub-add” surface and (b) “add” surface at the heat flux of *q''*~1900 kW/m^2^


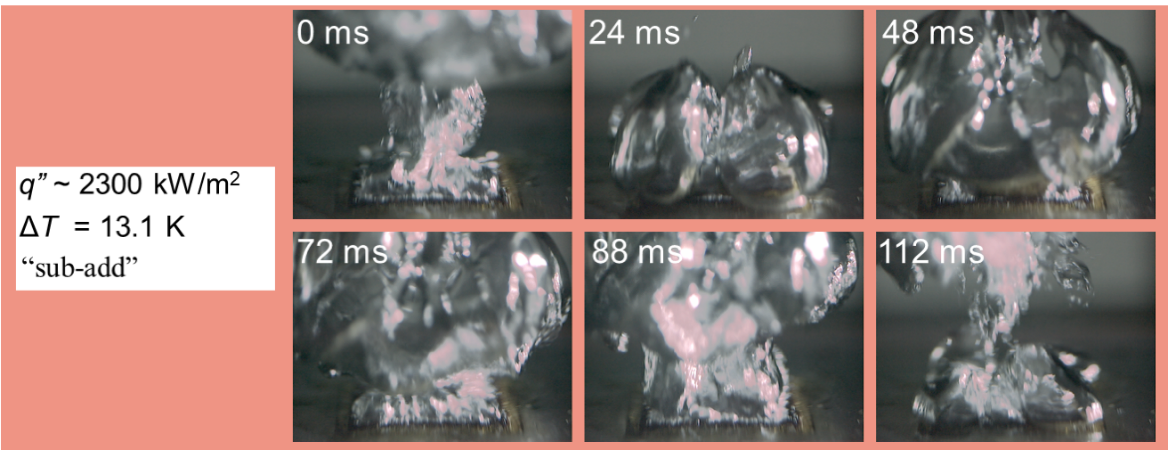


**Figure S33** Bubble images for the “sub-add” surface at the heat flux of *q''*~2300 kW/m^2^

**S10 Quenching experimental apparatus and experimental process**

The experimental setup is shown schematically in Fig. S34, which was mainly consisted of an electric actuator (LEFS16AB-200B-R16N, SMS Co. Ltd., Japan), a tube furnace (MTF 10/25/130, Carbolite Co. Ltd., UK), a quenching pool made of quartz glass, a heating platform (c-mag HS10, IKA Co. Ltd., Germany), a high-speed camera (GX-1, NAC Co. Ltd., Japan) and a data acquisition instrument (Agilent 34972A, Agilent Co. Ltd., USA). Readers who are interested in the detailed specifications are encouraged to refer to our previous work^[48]^.

The sphere samples were made of 304 stainless steel with a diameter of 10 mm. A hole with a diameter of 1.5 mm was drilled into each sphere sample for temperature measurement, allowing the insertion of a K-type thermocouple. Prior to every quenching test, deionized water in the pool was heated to the saturation temperature (100 ℃ at the atmospheric pressure) and held at the temperature for 30 min for degassing. Meanwhile, the modified samples were heated to 750 ℃ and then quickly dipped into the pool at a velocity of 25 cm/s using an electric actuator. The data acquisition system recorded the center temperature of the sphere at a frequency of 10 Hz. The high-speed camera was used to visualize the evolutions of the vapor films and bubbles at 500 frames per second.

In this work, the “add” samples were prepared by 6-step electrodeposition in electrolyte consisted of 0.2 M CuSO_4_ and 1 M H_2_SO_4_ at 25 ℃. The current density and deposition time were 3 A/cm^2^ and 10 s for every large-current step, the small-current step was the same with surfaces in S4. For the “sub-add” samples, the samples were firstly etched at 1 A/cm^2^ for 60 s, then following by the same deposition procedure with the “add” surfaces. After the samples were dried in air for 15 min, they were placed in the tube furnace at 300 ℃ for 30 min to strengthen the deposited structure before tests.

Under the assumption of one-dimensional heat conduction in spherical geometry, the surface-averaged heat flux (*q’’*_s_) over the sphere and surface temperature (*T*_s_) were estimated based on the center temperature (*T*_c_) of the sphere^[48]^. Solving the inverse heat conduction equation and truncating at the three leading terms of the series solution, one can obtain:

|  |  | (S16) |
| --- | --- | --- |
|  |  | (S17) |

where:

: density of 304 stainless steel, kg/m^2^.

*c*_p_: specific heat capacity of 304 stainless steel, J/(kg·K).

*R*: radius of sphere samples, m.

*T*_c_: center temperature captured by K-type thermocouple, K.

*α*: thermal diffusivity of 304 stainless steel, m^2^/s.


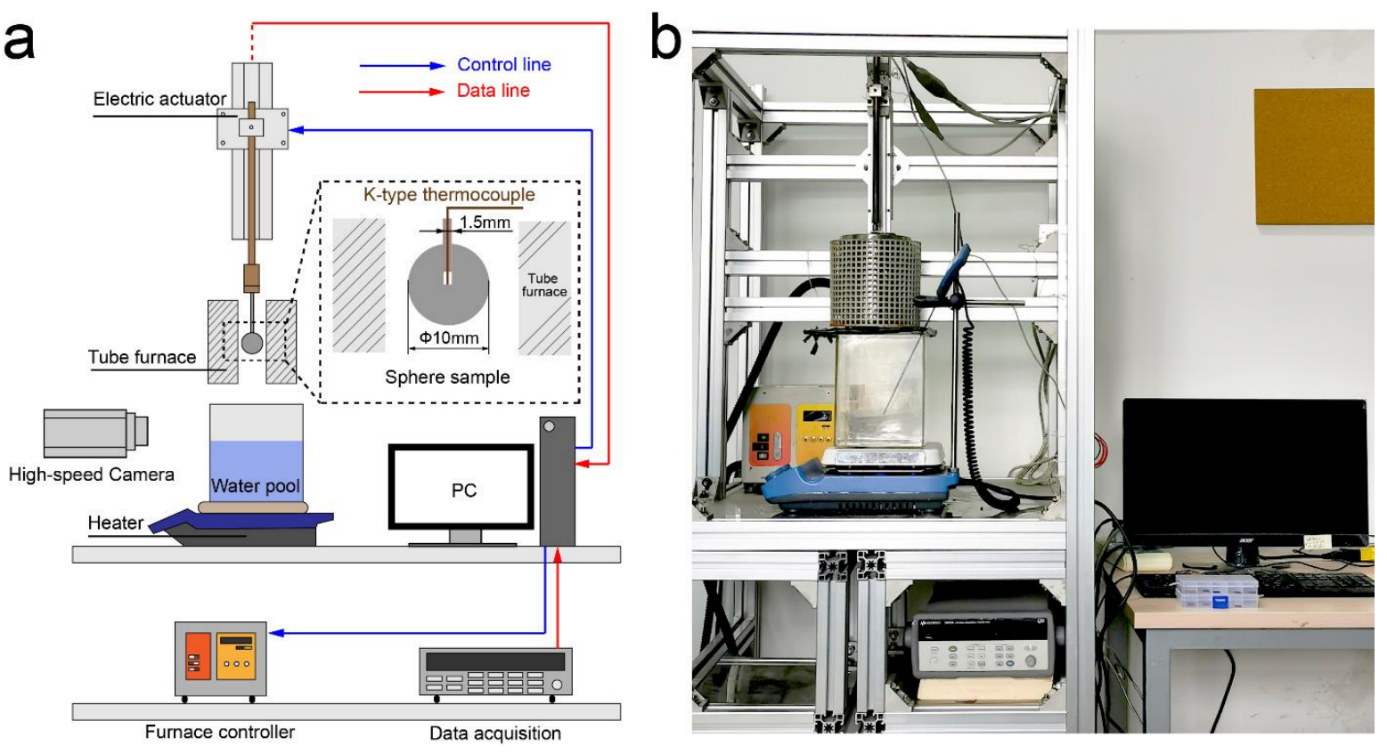


**Figure S34** Quenching boiling experimental setup: (a) schematic and (b) photograph

**S11 Videos**

**Video S1.** Water vapor bubble behavior on the “add” surface at *q''*~70 kW/m^2^

**Video S2.** Water vapor bubble behavior on the “sub-add” surface at *q''*~70 kW/m^2^

**Video S3.** Water vapor bubble behavior on the “add” surface at *q''*~1300 kW/m^2^

**Video S4.** Water vapor bubble behavior on the “sub-add” surface at *q''*~1300 kW/m^2^

**Video S5.** Water vapor bubble behavior on the “add” surface at *q''*~1900 kW/m^2^

**Video S6.** Water vapor bubble behavior on the “sub-add” surface at *q''*~1900 kW/m^2^

**Video S7.** Water vapor bubble behavior on the “sub” surface at *q''*~2300 kW/m^2^

**References**

1. Y. Song, C. D. Díaz-Marín, L. Zhang, H. Cha, Y. Zhao, E. N. Wang, *Adv. Mater*. **2022**, *34*, 2200899.
2. H. Tang, L. Xia, Y. Tang, C. Weng, Z. Hu, X. Wu, *Renew. Energy* **2022**, *187*, 790.
3. G. Choi, D. I. Shim, D. Lee, B. S. Kim, H. H. Cho, *Int. Commun. Heat Mass Transfer* **2019**, *109*, 104331.
4. X. Li, S. Wang, R. Wen, X. Ma, R. Yang, *Cell Rep. Phys. Sci* **2022**, *3*, 100746.
5. C. M. Patil, K. S. V. Santhanam, S. G. Kandlikar, *Int. J. Heat Mass Transfer* **2014**, *79*, 989.
6. Y.-Q. Wang, S.-S. Lyu, J.-L. Luo, Z.-Y. Luo, Y.-X. Fu, Y. Heng, J.-H. Zhang, D.-C. Mo, *Appl. Surface Sci.* **2017**, *422*, 388.
7. A. M. Gheitaghy, H. Saffari, D. Ghasimi, A. Ghasemi, *Appl. Therm. Eng.* **2017**, *113*, 1097.
8. J. Li, W. Fu, B. Zhang, G. Zhu, N. Miljkovic, *ACS Nano* **2019**, *13*, 14080.
9. S. Kalita, D. Sen, P. Sen, S. Das and B. B. Saha, *Int. Commun. Heat Mass Transfer* **2023**, *144*, 106740.
10. M. Hong, H. Lu, J. Luo, J. Gu, D. Mo, S. Lyu, Y. Heng, *Appl. Therm. Eng.* **2021**, *192*, 116809.
11. A. M. Gheitaghy, H. Saffari, M. Mohebbi, *Exp. Therm. Fluid Sci.* **2016**, *76*, 87.
12. A. M. Rishi, A. Gupta, S. G. Kandlikar, *Appl. Therm. Eng.* **2018**, *140*, 406.
13. A. M. Rishi, S. G. Kandlikar, A. Gupta, *Int. J. Heat Mass Transfer* **2019**, *132*, 462.
14. S. K. Gupta, R. D. Misra, *J. Therm. Anal. Calorim.* **2019**, *136*, 1781.
15. S. K. Gupta, R. D. Misra, *Int. Commun. Heat Mass Transfer* **2018**, *97*, 47.
16. D.-C. Mo, S. Yang, J.-L. Luo, Y.-Q. Wang, S.-S. Lyu, *Int. J. Heat Mass Transfer* **2020**, *157*, 119867.
17. A. M. Gheitaghy, H. Saffari, G. Q. Zhang, *Heat Transfer Eng.* **2019**, *40*, 762.
18. H. Shakeri, A. Heidary, H. Saffari, S. M. Hosseinalipoor, *Chem. Eng. Process* **2023**, *187*, 109296.
19. B. Shil, D. Sen, A. Kumar Das, P. Sen, S. Kalita, S. Das, *Therm. Sci. Eng. Prog.* **2023**, *43*, 101965.
20. H. Pandey, H. Mehrabi, A. Williams, C. Mira-Hernández, R. H. Coridan, H. Hu, *Appl. Therm. Eng.* **2024**, *236*, 121807.
21. D. Cooke and S. G. Kandlikar, *Int. J. Heat Mass Transfer* **2012**, *55*, 1004.
22. D. Zhong, J. a. Meng, Z. Li, Z. Guo, *Int. J. Heat Mass Transfer* **2015**, *87*, 201.
23. K.-H. Chu, R. Enright, E. N. Wang, *Appl. Phys. Lett.* **2012**, *100*, 241603.
24. A. M. Gheitaghy, A. Samimi, H. Saffari, *Appl. Therm. Eng.* **2017**, *126*, 892.
25. Y. Song, S. Gong, G. Vaartstra, E. N. Wang, *ACS Appl. Mater.*, **2021**, *13*, 12629.
26. A. Zou, D. P. Singh, S. C. Maroo, *Langmuir*, **2016**, *32*, 10808.
27. Z. Xu, X. Zhou, Y. Qiu, J. Xu, D. Shan, B. Guo, *Int. J. Heat Mass Transfer* **2024**, *220*, 124893.
28. W. Zhou, L. Mao, X. Hu, Y. He, *Carbon* **2019**, *150*, 168.
29. L. Mao, X. Hu, W. Zhou, *Carbon* **2020**, *164*, 184.
30. S. Das, D. S. Kumar, S. Bhaumik, *Appl. Therm. Eng.* **2016**, *96*, 555.
31. S. Das, R. Johnsan, C. S. Sujith Kumar, A. Datta, *J. Therm. Anal. Calorim.* **2021**, *144*, 1073.
32. X. Cheng, G. Yang, J. Wu, *Int. J. Heat Mass Transfer* **2022**, *192*, 122937.
33. X. Wang, J. Xu, H. Jiang, Y. Liu, X. Li, D. Shan, *Appl. Therm. Eng.* **2023**, *227*, 120441.
34. W. M. Rohsenow, *Trans. ASME*, **1952**, *74(6)*, 969.
35. S. G. Kandlikar, *J. Heat Transfer*, **2001**, *123(6)*, 1071.
36. W. M. Rohsenow, J. P. Hartnett, and Y. I. Cho, *Handbook of Heat Transfer* **1998**, 3, New York: McGraw-Hill.
37. L. Z. Zeng, J. F. Klausner, and R. Mei, *Int. J. Heat Mass Transfer* **1993**, *36*, 2261.
38. N. Zuber. *Int. J. Heat Mass Transfer* **1961**, *2*, 83.
39. C. E. Brennen, *Cavitation and Bubble Dynamics*, **2014**, Cambridge University Press.
40. Y. Y. Hsu, *J. Heat Transfer*, **1962**, *84*, 207.
41. G-Y. Su, C. Wang, L. Zhang, Jee Hyun Seong, R. Kommajosyula, B. Phillips, and M. Bucci, *Int. J. Heat Mass Transfer* **2020**, *160*, 120134.
42. R. L. Mohanty, M. K. Das, *Renew. Sust. Energ. Rev.* **2017**, *78*, 466.
43. M. M. Mahmoud, T. G. Karayiannis, *Therm. Sci. Eng. Prog.* **2021**, *25*, 101023.
44. M. M. Rahman, E. Ölçeroğlu, M. McCarthy, *Langmuir* **2014**, *30*, 11225.
45. K.H. Chu, R. Enright, E. N. Wang, *Appl. Phys. Lett.* **2012**, *100*, 241603.
46. J. Li, J. Zhang, L. Mou, Y. Zhang, L. Fan, *Int. J. Heat Mass Transfer* **2019**, *141*, 835.
47. J. Zhang, L. Fan, J. Li, Z. Yu, *Int. J. Heat Mass Transfer* **2020**, *162*, 120364.
48. L. Fan, J. Li, D. Li, L. Zhang, Z. Yu, *Int. J. Heat Mass Transfer* **2014**, *76*, 81.
